# Genome-Guided Analysis and Whole Transcriptome Profiling of the Mesophilic Syntrophic Acetate Oxidising Bacterium *Syntrophaceticus schinkii*

**DOI:** 10.1371/journal.pone.0166520

**Published:** 2016-11-16

**Authors:** Shahid Manzoor, Erik Bongcam-Rudloff, Anna Schnürer, Bettina Müller

**Affiliations:** 1 Department of Information Technology, University of the Punjab, Lahore, Pakistan; 2 Department of Animal Breeding and Genetics, Swedish University of Agricultural Science, SLU-Global Bioinformatics Centre, Uppsala, SE 750 07, Sweden; 3 Department of Microbiology, Swedish University of Agricultural Sciences, BioCenter, Uppsala, SE 750 07, Sweden; Westfalische Wilhelms-Universitat Munster, GERMANY

## Abstract

*Syntrophaceticus schinkii* is a mesophilic, anaerobic bacterium capable of oxidising acetate to CO_2_ and H_2_ in intimate association with a methanogenic partner, a syntrophic relationship which operates close to the energetic limits of microbial life. *Syntrophaceticus schinkii* has been identified as a key organism in engineered methane-producing processes relying on syntrophic acetate oxidation as the main methane-producing pathway. However, due to strict cultivation requirements and difficulties in reconstituting the thermodynamically unfavourable acetate oxidation, the physiology of this functional group is poorly understood. Genome-guided and whole transcriptome analyses performed in the present study provide new insights into habitat adaptation, syntrophic acetate oxidation and energy conservation. The working draft genome of *Syntrophaceticus schinkii* indicates limited metabolic capacities, with lack of organic nutrient uptake systems, chemotactic machineries, carbon catabolite repression and incomplete biosynthesis pathways. Ech hydrogenase, [FeFe] hydrogenases, [NiFe] hydrogenases, F_1_F_0_-ATP synthase and membrane-bound and cytoplasmic formate dehydrogenases were found clearly expressed, whereas Rnf and a predicted oxidoreductase/heterodisulphide reductase complex, both found encoded in the genome, were not expressed under syntrophic growth condition. A transporter sharing similarities to the high-affinity acetate transporters of aceticlastic methanogens was also found expressed, suggesting that *Syntrophaceticus schinkii* can potentially compete with methanogens for acetate. Acetate oxidation seems to proceed via the Wood-Ljungdahl pathway as all genes involved in this pathway were highly expressed. This study shows that *Syntrophaceticus schinkii* is a highly specialised, habitat-adapted organism relying on syntrophic acetate oxidation rather than metabolic versatility. By expanding its complement of respiratory complexes, it might overcome limiting bioenergetic barriers, and drive efficient energy conservation from reactions operating close to the thermodynamic equilibrium, which might enable *S*. *schinkii* to occupy the same niche as the aceticlastic methanogens. The knowledge gained here will help specify process conditions supporting efficient and robust biogas production and will help identify mechanisms important for the syntrophic lifestyle.

## Introduction

Large-scale production of bio-methane through anaerobic degradation (AD) of organic matter is an alternative sustainable energy source suitable for replacing fossil vehicle fuels and for delivering heat and electricity. Many European countries envisage bio-methane as the means to increase the amount of renewable energy in order to meet the European Union 20-20-20 goals (http://www.iea-biogas.net/country-reports.html).

In order to operate biogas plants economically and avoid competition with food and feed production, interest in using alternatives to energy crops has grown dramatically. In particular, protein-rich feedstocks such as slaughterhouse waste, distiller’s grain and organic food waste are receiving great attention, since they have high methane yield potential and result in a biogas digestion residue that is rich in plant-available ammonium. However, when proteinaceous materials are used, ammonia is released continuously and this has a direct impact on the prevailing methane production pathway, with consequences for process stability and efficiency [1–3].

Acetate, formate, H_2_ and CO_2_ are the main intermediate products of AD and the methanogenic substrates [4]. Two mechanisms for acetate conversion to methane have been described: Aceticlastic methanogenesis performed by members of the genera *Methanosarcina* and *Methanosaeta*, which comprises direct cleavage of acetate to methane and CO_2_ [5, 6], and syntrophic acetate oxidation (SAO), performed by intimate cooperation between acetate-oxidising bacteria (SAOB) and H_2_/formate-consuming methanogens [7–9]. The direct consumption of H_2_ by hydrogenotrophic methanogens drives the thermodynamically unfavourable acetate oxidation:

$$\begin{array}{l} CH_{3}COO^{-}+H^{+}+2H_{2}O\to 2CO_{2}+4H_{2}△G^{o`}=+95\;kJ\;per\;mol\;rct.\\ 4H_{2}+CO_{2}\to CH_{4}+2H_{2}O\;△G^{o`}=-131\;kJ\;per\;mol\;rct.\\ CH_{3}COO^{-}+H^{+}+\to CH_{4}+CO_{2}△G^{o`}=-35\;kJ\;per\;mol\;rct. \end{array}$$

Aceticlastic methanogenesis is thermodynamically more favourable [10], but is strongly inhibited by high ammonia concentrations [11, 12], ceding the advantage to the less thermodynamically favourable SAO [13–15]. Other factors such as dilution rate, acetate concentration, methanogenic population and CO_2_ partial pressure have also been shown to influence the methanogenic pathway from acetate [16–23].

Syntrophic acetate oxidation (SAO) has been identified in constructed biogas reactors [14, 15, 19, 24, 25], but also in a wide range of natural anaerobic environments such as rice paddy soil, subtropical lake sediment, oil reservoirs and nutrient-enriched soils [21, 26–31]. This emphasises the dual nature of this process as a controlled waste treatment contributing to renewable ‘green’ energy production on the one hand, and as a potential driver of greenhouse gas emissions from natural habitats on the other.

The phylogenetically diverse SAOB are poorly understood and strict cultivation requirements and difficulties in reconstituting the thermodynamically unfavourable SAO process under laboratory conditions prevent comprehensive investigation of their metabolism. Only two thermophilic (*Pseudothermotoga lettingae*, *Thermacetogenium phaeum*) and three mesophilic representatives (*Tepidanaerobacter acetatoxydans*, *Clostridium ultunense*, *Syntrophaceticus schinkii*) have been characterised [32–36], all isolated from engineered biogas processes. Heterotrophic cultivation experiments and enzyme activity studies affiliate the majority of the SAOB to the physiological group of acetogens [37, 38], which have the Wood-Ljungdahl pathway (WL) as a common metabolic feature. Two possible pathways have been suggested for SAO: i) Enzyme activity studies using crude cell extract and genome analysis indicate involvement of the reversed WL pathway in syntrophic acetate oxidation in the case of *T*. *phaeum* and *C*. *ultunense* [37, 39, 40]. In the case of *T*. *acetatoxydans*, the genome harbours a truncated WL pathway, organised in one operon, but the lack of ATP synthase does not favour the use of a reversed WL pathway [41, 42]. ii) An alternative pathway is supposed to circumvent the carbonyl branch of the WL pathway by combining the glycine cleavage system with the methyl branch of the WL pathway, as suggested for a terephthalate-degrading *Mesotoga* community, and the thermophilic SAOB *P*. *lettingae* [43], however more experimental data are needed to further support this route.

In the case of *S*. *schinkii* very less is known about the metabolic machinery employed for syntrophic acetate oxidation. A previous genetic study revealed the presence and expression of the formyltetrahydrofolate synthetase gene, however this is a key enzyme of both suggested SAO pathways [44]. However, very recently a draft genome sequence of *S*. *schinkii* became available [45]. Therefore, the aim of the present study was to reveal metabolic features related to SAO, energy conservation and syntrophic interactions of the mesophilic SAOB *S*. *schinkii*, the most abundant and enduring SAOB found in high-ammonia and also low-ammonia mesophilic large-scale and laboratory-scale biogas processes [15, 25, 46] by performing genome-guided analysis of physiological and metabolic traits and transcriptome profiling of SAO co-cultures using next-generation sequencing (RNA seq).

## Materials and Methods

### Genome sequencing, annotation and analysis of physiological and metabolic capacities

Cell growth conditions and isolation of total DNA were as described by [35]. The genome of *S*. *schinkii* was sequenced at the SciLifeLab Uppsala, Sweden, using Ion Torrent PM systems with a mean length of 206 bp, longest read length 392 bp and a total of final library reads of 2,985,963 for single end reads. Information about genome sequencing and assembly, genome annotation and genome properties such as number of contigs and scaffolds, sequencing coverage, and gap closing information are described in detail in [45]. All CDSs predicted by available tools in the Magnifying Genome (MaGe) pipeline were translated and used to search the National Center for Biotechnology Information (NCBI) non-redundant database and the UniProt, TIGRFam, Pfam, PRIAM, KEGG, COG and InterPro databases using the Basic Local Alignment Search Tool for Proteins (BLASTP). Manual searches and annotation were performed using the same tools in MaGe [47]. The transporter database (TCDB;http://www.tcdb.org) [48] was used to identify all transporters in the genome of *S*. *schinkii*. Twin-arginine transport signal sequences were predicted by the TatP server at http://www.cbs.dtu.dk/services/TatP [49]. Identification numbers given in the text for individual genes are MaGe locus tag numbers, which can be used to search for genes on the MaGe website. Comparative analysis of *S*. *schinkii* Sp3 and *T*. *phaeum* was performed using a set of tools available in EDGAR (Efficient Database framework for comparative Genome Analyses using BLAST score Ratios) [50].

### Transcriptomic analysis

mRNA was purified from three acetate-oxidising co-cultures including *S*. *schinkii* Sp3 and *Methanoculleus bourgensis* MAB1, after 30 to 50% of the initially added 100 mM acetate was consumed. Medium preparation and cultivation conditions were as described by [8, 42]. 3g/L ammonium chloride were added. At first, total RNA was purified using the ZR Soil/Fecal RNA Kit from Zymo Research (Irvine, CA, USA) according the manufacturer’s instructions with the following modifications: The lysis buffer was replaced by 1 mL TRizol® reagent (Ambion, Thermo Fisher Scientific, Waltham, MA, USA) and 0.2 mL chloroform. The respective centrifugation step was extended to 10 min at 4°C. Depletion of ribosomal RNA was conducted using Ribo-Zero rRNA Removal Kit for bacteria (Illumina, San Diego, CA, USA) following the manufacturer’s manual. Quantity and quality of total RNA and depleted RNA samples were assessed using a Bioanalyzer 2100 (Agilent Technologies, Santa Clara, CA, USA). Single-end sequencing was performed by Uppsala Genome Center (Uppsala, Sweden) using Ion Proton technology in duplicates. Raw 85-bp (mean read length) RNA-seq reads were mapped to the working draft genome of *S*. *schinkii* strain Sp3 (CDRZ01000000) using STAR 2.5 [51]. Each mapped read was associated with an ENSEMBL gene. htseq-count script was used to count the number of reads mapped to each gene/feature [52]. After raw data quality control and pre-processing, the total number of reads from triplicated co-cultures and technical duplications were 2,4364,534, 9,280,101, 32,281,907, 23,596,991, 23,588,408, and 22,665,529, respectively. Thereof 7,294,53 (2.99%), 1,515,15 (1.63%), 17,218,75 (5.33%), 13,966,61 (5.92%), 2,838,00 (1.20%), 3,354,74 (1.48%) number of reads could be mapped against *S*. *schinkii* genome. Gene counts were length normalized and the FPKM values (fragments per kilobase of transcript per million mapped reads, log2 expression) relatively to a housekeeping (HK) gene (gyrA) were calculated. FPKM values are represented as mean values with standard deviation. The sequencing data obtained were submitted to ArrayExpress and have been affiliated to accession number E-MTAB-4310.

## Results and Discussion

### Phenotypic features of *Syntrophaceticus schinkii*

Sporulation, oxidative stress response, motility and chemotaxis mediate flexibility to changing environmental conditions, oxygen traces and nutrient depletion. *S*. *schinkii* might have the ability to tolerate small amounts of oxygen, since besides manganese catalase and rubrerythrin encoding genes [45] the genome harbours a superoxide dismutase gene (SSCH_220034). It has also been shown to survive starvation and environmental stress by forming endospores [35]. A total of 38 genes were assigned to sporulation-specific functions (S1 Table) including the master regulator Spo0A, and the sporulation-specific sigma factors SigE, K and F [45]. In contrast to other SAOB, *S*. *schinkii* appears to be restricted in chemotactic manoeuvres due to lack of any flagellum-related genes and the basic chemotaxis machinery CheA/CheY [reviewed in [53]]. However, we found evidence in the genome that *S*. *schinkii* is potentially able to move by gliding using fimbrial structures, since the genome encodes a type IV pilus apparatus consisting of PilC/T/B/D/M and FimT (SSCH_700002–19; S1 Table). Type IV pili mediate twitching and gliding motilities (reviewed in [54]) by generating a retractable force performed by the ATPase PilT, which enables the cells to move [55, 56]. Type IV pilus retraction is also indispensable for biofilm formation and transformation and is related to phage sensitivity. The PilB/PilC operon (SSCH_700018–19) encoding the inner membrane core protein and the assembly ATPase was found to be clearly expressed under SAO conditions (S1 Fig). A putative second cluster including a PilT homologue (SSCH_60043–51; S1 Table) was predicted elsewhere in the genome, which genes were partly expressed too (S1 Fig). It has also been shown that pili have a direct role in electron transfer for *Geobacter* species by forming microbial nanowires from a protein subunit that has high homology to the type IV protein, pilA [57]. In *S*. *schinkii*, two ORFs have been predicted as putative type IV pilin PilA family proteins with the conserved amino-terminal amino acid characteristics of type IV pilins (SSCH_1170015, SSCH_700017). The identities of these ORFs to the *Geobacter* homologs are below 30%, but they also share less sequence coverage and identity to pilin related proteins in general as it is the case for *Geobacter* pilA homologs (Blastp, [58]).

A further unique trait, which has not been reported for other SAOB, is the potential ability to perform quorum sensing (QS) using a LuxI/LuxR-type QS circuit that expresses and monitors acylated homoserine lactones, also called autoinducer 1, which is usually found in Gram-negative bacteria (reviewed in [59]). Acyl-homoserine-lactone synthase LuxI (SSCH_1110008), at least three LuxR-related transcriptional regulator (SSCH_1220017, SSCH_170030, SSCH_170036) and two acyl-carrier proteins (SSCH_1110009, SSCH_190038), which deliver acyl groups to the synthase, were found encoded in the genome. Bacteria use QS to track changes in their cell numbers and collectively alter gene expression, which enables cooperative behaviour correlated to virulence, biosynthesis of secondary metabolites and biofilm formation [59]. *S*. *schinkii* might use this trait to coordinate activities that are beneficial when performed together, such as attracting the methanogenic partner, forming flocs and/or biofilms and synchronising metabolism in order to initiate syntrophy. Two of the putative LuxR-related transcriptional regulators (SSCH_170030, SSCH_170036) as well as both of the acyl-carrier proteins (SSCH_1110009, SSCH_190038) were found to be expressed (S1 Fig). A weak expression was found for the acyl-homoserine-lactone synthase LuxI (SSCH_1110008, S1 Fig).

Corresponding to the moderate growth temperatures (between 25 and 40°C), the genome encodes the heat shock proteins Hsp20 (SSCH_540016, SSCH_1060017), GrpE (SSCH_170005), GroEL and GroES (SSCH_160020/21; SSCH_1380009/10/11) and a Clp protease (SSCH_80029/30). *T*. *acetatoxydans*, which can cope with temperatures up to 55°C, and *T*. *phaeum*, which grows between 40 and 65°C, have with seven [40] and eight [42] different Hsp genes (GroL, GroS, GroEL, DnaJ, DnaK, ClpB, GrpE and Hsp20), respectively, a comparatively higher number. Heat shock proteins Hsp20 (SSCH_1060017), GroES (SSCH_160020/21; SSCH_1380009/10/11), GroEL (SSCH_160020/21), and the Clp protease (SSCH_80029/30) were found to be part of the stress response under syntrophic growth conditions at 37°C (S2 Fig).

**Fig 1:**
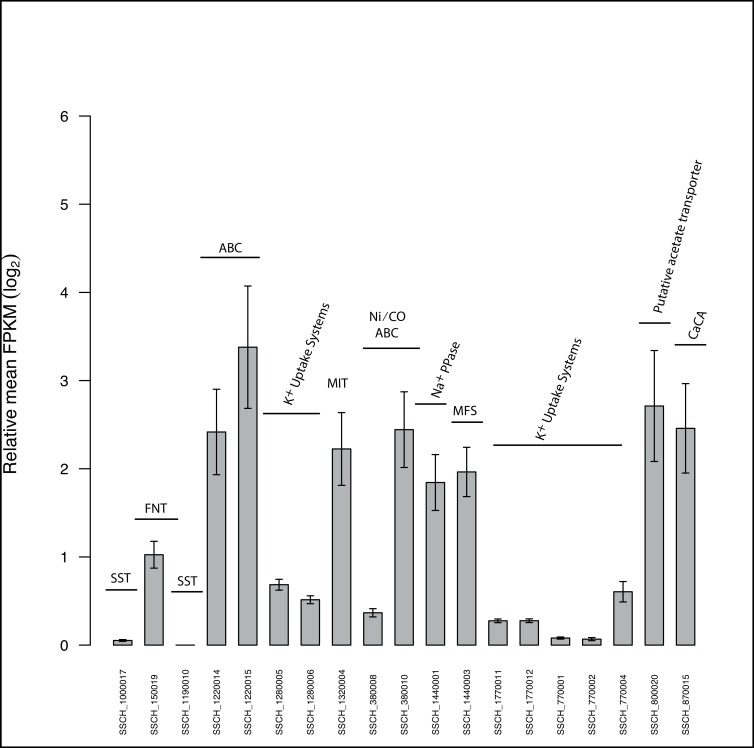
Bar graph showing the FPKM values of genes related to transport systems. SST, solute sodium transporter; FNT, formate/nitrite transporter; MIT, metal ion transporter; MFS, major facility superfamily transporter; CaCA, Ca^2+^/cation antiporter; Na+PPase, Na^+^ pyrophosphate energised pump; ABC, ATP-binding cassette transporters.

Another characteristic of the SAOB is their extremely high tolerance to ammonia [38, 46]. This ammonia resistance has been suggested to be the most selective factor for establishing SAO, due to the intrinsic osmosensitivity of aceticlastic methanogens to ammonia [11, 12]. *S*. *schinkii*, *T*. *acetatoxydans* and *C*. *ultunense* have even been shown recently to tolerate free ammonia concentrations up to 1 g/L in a gradually adapted laboratory-scale reactor [60]. A previous genome-scale analysis predicted five potential mechanisms preventing NH_4_^+^/NH_3_-induced osmotic stress in the case of *T*. *acetatoxydans* [42]. These included I) a common adaptive response as known for Gram-positive bacteria [61] involving rapid potassium uptake through potassium channels followed by II) accumulation of a compatible solute such as betaine, proline or glutamate, III) individual characteristics such as the lack of ammonium transporters and IV) the lack of the high affinity GS/GOGAT (glutamine synthetase/glutamate synthase) machinery for ammonium assimilation and V) the presence of potential Na+/H+ antiporters and V-type ATPases. A similar genotype was found for *S*. *schinkii*: Genes coding for two putative potassium uptake proteins (SSCH_1280005–6; SSCH_1770011–12; S2 Table), and two betaine/carnitine/cholin transporters (SSCH_450002; SSCH_450006) and one betaine/glycine ABC transport system (SSCH_560019–23; S2 Table) might enable an adaptive response. However, none of these uptake proteins was found expressed under the conditions analysed (Fig 1). Instead, four transporters sharing identities with a MFS (major facility superfamily) transporter (SSCH_1440003), a Ca^2+^/cation antiporter (SSCH_870015), a Na^+^ pyrophosphate energised pump (SSCH_1440001) and an unclassified ABC transport system (SSCH_1220014–15) were expressed and might play a role in osmotic stress response (Fig 1). As in *T*. *acetatoxydans*, the genome of *S*. *schinkii* lacks glutamine synthetase and putative ammonium transporters (BlastP search using reference sequences of the Amt/MEP/Rh ammonium transporter family [62]). The absence of glutamine synthetase gene might prevent a depletion of ATP, as expression of the low NH_4_^+^ affinity glutamate dehydrogenase, that is present in the genome of both *T*. *acetatoxydans* and *S*. *schinkii* (SSCH_1640002) does not function of the expense of ATP [42]. It has also speculated for *T*. *acetatoxydans* that the dependence on amino acid rich environments, might support a glutamate dehydrogenase function in detoxification rather than in ammonium assimilation [42]. The absence of ammonium transporters might protect the cells from redundant ammonium influx and might also explain why *S*. *schinkii* likewise to *T*. *acetatoxydans* has not been detected in ammonium–limited environments. A similar genotype has been described for the methanogenic partner organism *Methanoculleus bourgensis* MAB1 and for the type strain *M*. *bourgensis* MS2, whose genomes also lack genes related to ammonium transporter, but encode diverse potassium and osmolyte uptake systems [63, 64]. V-type ATPases suggested to support *T*. *acetatoxydans* in maintaining pH homeostasis [42] were not found in the genome of *S*. *schinkii*.

### Metabolic features of *Syntrophaceticus schinkii*

**Fig 2:**
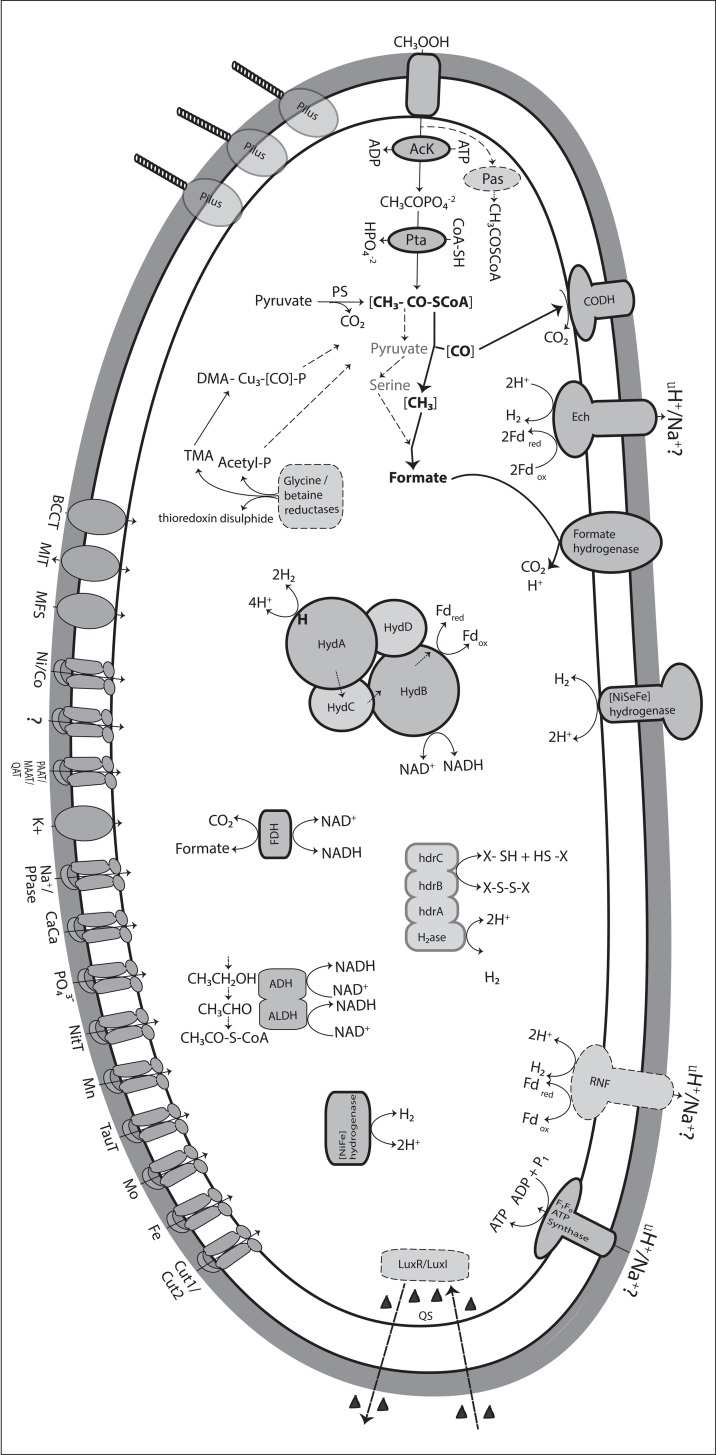
Overview of the predicted metabolism of *S*. *schinkii*. Bold shaped metabolic features were found expressed under acetate oxidising growth condition. CODH, carbon monoxide dehydrogenases; FDH, formate dehydrogenase; Rnf, H^+^/Na^+^? ferredoxin-NAD:oxidoreductase; Ech, energy-conserving hydrogenase; QS, quorum sensing; Ack, acetate kinase; Pta, phosphoacetyl transferase; Pas, predicted acetyl-CoA synthase (ADP-forming); ADH, alcohol dehydrogenase; ALDH, acetaldehyde dehydrogenase; CUT1/CUT2, carbohydrate uptake transporters; TauT, taurine uptake transporter family; NitT, nitrate/nitrite/cyanate2 uptake transporter family; PO_4_^3-^, phosphate uptake system; CaCa, Ca2+:cation antiporter family; Na+, sodium transporter; Na^+^/PPase, Na^+^ pyrophosphate energised pump; K^+^, potassium transporter family; PAAT, HAAT, amino acide uptake 1 transporter family; QAT, quaternary amine 1 uptake transporter family;?, Unclassified ABC-type transporter; MFS, major facility superfamily; MIT, metal ion transporter; BCCT, betaine/carnitine/choline transporter family; ABC transport systems for trace elements (Ni, Co, Mn, Mo, Fe).

*S*. *schinkii* obviously lacks active organic nutrient uptake systems, which could explain the extremely narrow substrate spectrum observed [38] and also indicates a very specialised metabolism (Fig 2).

Altogether, the genome contains 123 genes affiliated to 65 potential transport systems (S2 Table). A noteworthy finding was that apart from a few ion/solute transporters, only ATP binding cassette (ABC) transport systems are predicted to shuffle solutes across the membrane. *S*. *schinkii* does not harbour genes related to tripartite ATP-independent transporter (TRAP) or to the sugar phosphoenolpyruvate:phosphotransferase system (PTS), although both have been found in the SAOB *T*. *acetatoxydans* in high numbers [42]. The ABC transport systems are predicted to mainly transport trace elements such as Ni, Co, Mn, Zn, Mo and Fe, as well as amino acids (Fig 2, S2 Table). Only three of these are potential carbohydrate uptake systems. The predicted Ni/Co ABC transporter (SSCH_38008–10) and a putative metal ion transporter (MIT family, SSCH_1320004) were found expressed in the syntrophic co-culture (Fig 1) and might be involved in providing metal ions as cofactor to hydrogenases and carbon monooxide dehydrogenase.

*S*. *schinkii* strain Sp3 has been isolated as a heterotrophic organism utilising fermentation end products such as ethanol, lactate and betaine by forming acetate [38]. The genome harbours all enzymes needed for a functional WL pathway (see also detailed description in the section “Acetate oxidation”). Therefore, ethanol, betaine and lactate fermentation to acetate can potentially be linked to CO_2_ reduction via the WL pathway, as has been observed for the acetogens *Clostridium formicoaceticum* and *Acetobacterium woodii* when utilising lactate and ethanol, respectively [65, 66]. However, *S*. *schinkii* needs several months for doubling the cell number [38], whereas the reported doubling time for *C*. *formicoaceticum* on lactate and for *A*. *woodii* on ethanol is 5 h and 10 h, respectively. Ethanol degradation most likely proceeds via acetaldehyde using NAD^+^-dependent acetaldehyde and ethanol dehydrogenases (SSCH_320003, SSCH_1440007, SSCH_410009, SSCH_1120010) producing acetyl-CoA (Fig 2). Lactate degradation seems not to proceed via lactate dehydrogenase activity, since no ORF was predicted to encode this function. Pyruvate synthase (product of SSCH_330012–14, SSCH_480001–3) and pyruvate:formate lyase (product of SSCH_870024–25) for converting pyruvate to acetyl CoA and CO_2_ are present. One of the two clusters coding for the putative selenocysteine-containing glycine/betaine reductases [45] was found expressed (S3 Fig). These enzymes are probably responsible for uptake and conversion of betaine to acetylphosphate, thioredoxin disulphide and trimethylamine (TMA), when growing on betaine. The odorous, harmful TMA is a prominent by-product in the manufacture of fishmeal and has been suggested to be a product of microorganisms utilising choline, betaine and TMA N-oxide [67]. Since *S*. *schinkii* has been isolated from an anaerobic filter treating wastewater from a fishmeal factory [35], it might actively contribute to TMA formation. On the other hand, we found numerous genes and gene clusters dispersed in the genome related to TMA degradation, as described previously for *Methanosarcina* species [68, 69]. These include genes coding for trimethylamine: corrinoid and dimethylamine:corrinoid methyltransferases, corrinoid-binding proteins and methyltransferases (S3 Table). Encoding of the methyltransferase genes of *M*. *barkeri* require the synthesis and incorporation of pyrrolysine. The genome of *S*. *schinkii* harbours a putative *pylS* gene (SSCH_980007) that codes for pyrrolysyl-tRNA synthetase and putative pyrrolysine synthesis genes *pylBCD* (SSCH_980006–10) (S1 Table). These gene sets might allow TMA degradation and/or the formation of compatible solute such as betaine through a link by corrinoid-binding proteins to enzymes belonging to the WL pathway. None of the methyltransferases or corrinoid-binding proteins appears to be involved in the SAO pathway (S3 Fig).

The sugar utilisation capacities found in the genome might be employed in anabolic pathways providing precursors for biosynthesis, rather than being used for ATP generation. Although the genome encodes all the enzymes needed for expression of the Embden-Meyerhof-Parnas pathway, organised in three clusters (S4 Table), no growth has been reported on glucose or any other sugar or sugar derivative [38]. This can probably be explained by the lack of sugar PTS and the restricted number of predicted carbohydrate ABC transport systems, as mentioned above, as well as the lack of genes related to the Entner-Doudoroff pathway and the oxidative branch of the pentose phosphate pathway. As a further adaptation to the specialist syntrophic lifestyle, the genome of *S*. *schinkii* and that of *T*. *phaeum* both seem to lack genes related to carbon catabolite repression (CCR), such as catabolite gene-activator protein (CAP), adenylate cyclase and histidine protein (HPr), which usually confer competiveness in natural environments. In contrast, *T*. *acetatoxydans* harbours all genes needed for CCR, but also has a slightly broader substrate spectrum [42]. All genes needed for gluconeogenic enzyme activities, such as SSCH_630024 (pyruvate carboxylase), SSCH_180001 (pyruvate-phosphate dikinase), and SSCH_790022 (fructose-1,6 bisphosphatase), were expressed in *S*. *schinkii* (S3 Fig).

### Acetate oxidation

**Fig 3:**
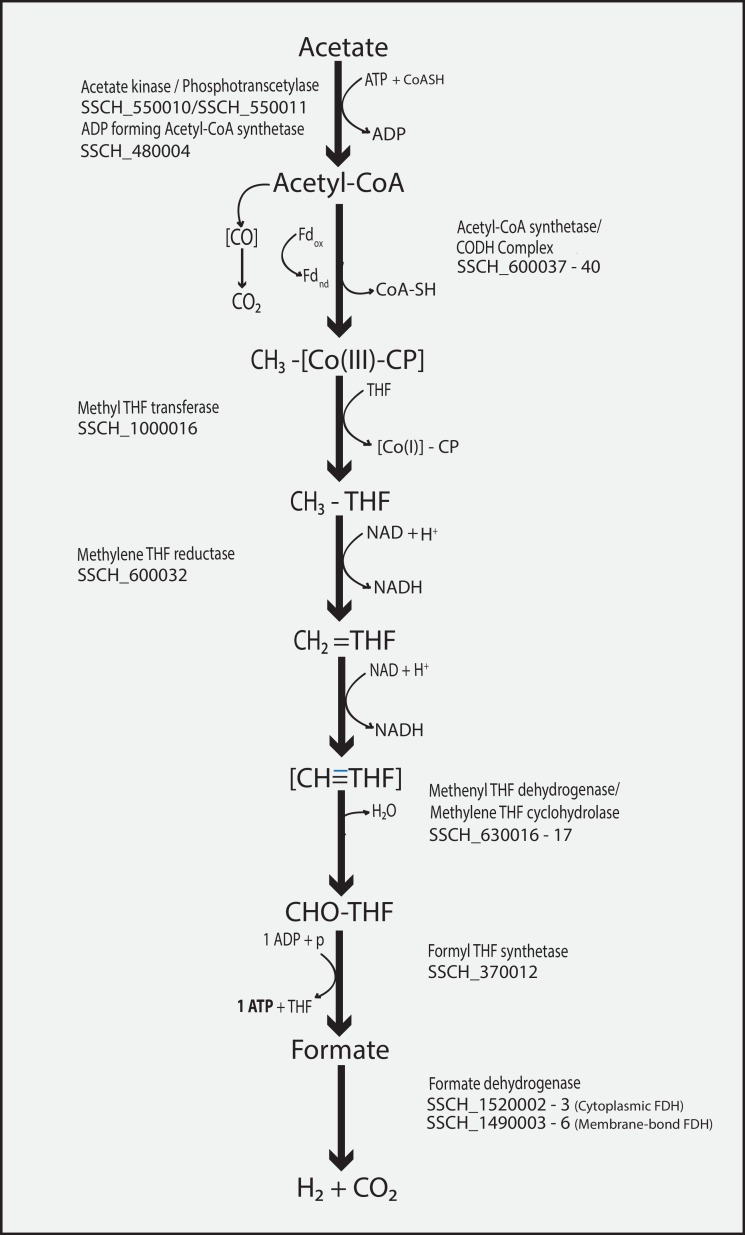
Oxidative Wood-Ljungdahl pathway of *S*. *schinkii* strain Sp3. THF (tetrahydrofolate), Co(III)/Co(I)-CP (corrinoid protein).

In our recent genome-scale analysis of the mesophilic SAOB *T*. *acetatoxydans* [42], we expressed doubts regarding the use of the reverse WL pathway, based on the lack of key enzymes such as formate dehydrogenase and F_1_F_0_-ATP synthase. As the only potential acetate-oxidising pathway generating net ATP, we identified a potential oxidative tricarboxylic acid cycle, as suggested for the sulphate-reducing bacteria *Desulfobacter postgatei* and *Desulfobacter hydrogenophilus* [70, 71]. This pathway can be excluded in the case of *S*. *schinkii* due to the lack of key enzymes such as succinyl-CoA transferase or citrate lyase. However, *S*. *schinkii* can potentially use both the oxidative direction of the WL pathway and the alternative route consisting of a combination of glycine cleavage pathway and WL pathway, as suggested by Nobu et al. [43], since the genome encodes all enzymes and proteins needed (Fig 3, S5 Table).

Most of the genes of the WL pathway are encoded once, except for formate dehydrogenases (FDH) and carbon monoxide dehydrogenases (CODH), which were found encoded at two loci (S5 Table). One *fdh* gene cluster (SSCH_1520002–1520003) was found to be flanked by genes coding for a putative molybdenum ABC transport system (S2 Table). A second locus (SSCH_1490003–1490006) includes a potentially associated cytochrome b subunit gene (SSCH_1490004) and most likely encodes a membrane-associated FDH. It shares the highest identities (56–74%) with the FDH of sulphate reducers and the syntrophic fatty acid oxidiser *Syntrophomonas wolfei* [72]. For both the presence of two or more FDH has been reported, whereas the individual expression depends on the trophic level occupied and is strongly regulated by an antagonistic effect of Mo and W [73, 74]. The thermodynamically unfavourable fatty acid oxidation strongly relies on interspecies H_2_ transfer but also a major involvement of formate has been proposed [75]. If electron-conducting pili are involved (section “Phenotypic features of *S*. *schinkii*) still needs to be addressed.

**Fig 4:**
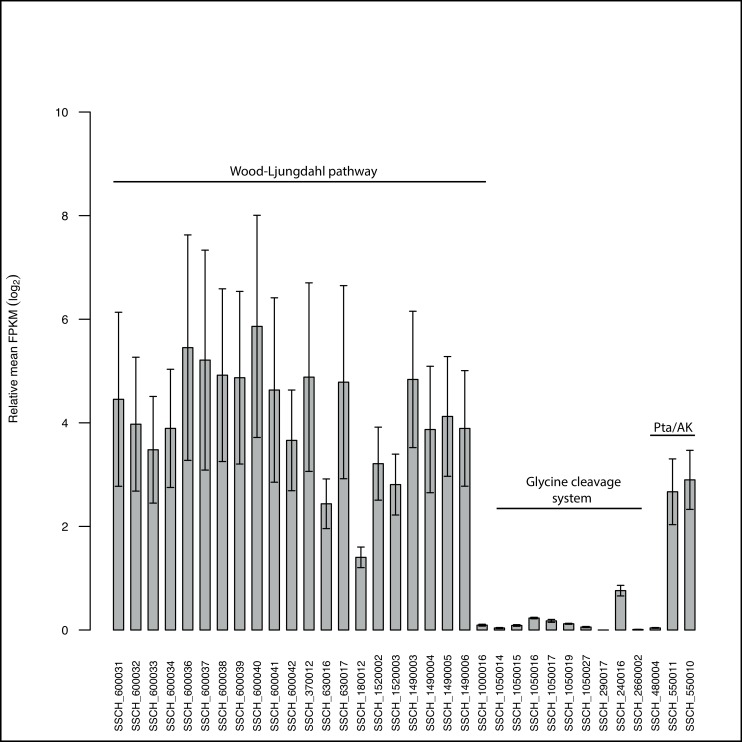
Bar graph showing the FPKM values of genes related to predicted SAO pathways. Pta, phosphoacetyl transferase; AK, acetate kinase.

One of the CODHs is part of the bifunctional CODH/acetyl-CoA synthase complex (SSCH_600040–600041), forming acetyl-CoA from a carbonyl group, a methyl group and CoA. The putative operon (SSCH_600031–600042; S4 Fig, S5 Table) also contains a 5,10-methylene-tetrahydrofolate (THF) reductase (SSCH_600032) and two genes resembling heterodisulphide reductase-like genes (SSCH_600031,600034). The second CODH (SSCH_180012) is located separately and shows 67% and 68% identity to the proton-translocating CODHs of *Methanosarcina barkeri* and *Methanosarcina mazei*, respectively. Both of the CODH as well as the heterodisulphide reductase-like genes were expressed, indicating importance in electron transport and proton translocation (Fig 4).

Except for 5,10-methylene-THF reductase, genes belonging to the methyl branch of the WL pathway were found elsewhere in the genome, away from the operon described above (S5 Table), including formyl-THF synthetase (SSCH_370012), methylene-THF dehydrogenase/methenyl-THF cyclohydrolase (SSCH_630016/17), a second methyl transferase (SSCH_1000016) and FDH as described above. The existence of only one formyltetrahydrofolate synthetase gene [44] was confirmed, whereas *T*. *phaeum* and *T*. *acetatoxydans* both harbour two *fhs* genes [40, 42]. The gene structure of the operon designated *acs* is identical to that found in the thermophilic SAOB *T*. *phaeum*, but different from that found in *T*. *acetatoxydans* (S4 Fig). All genes associated with the WL pathway were clearly expressed, whereas genes coding for functions employed by the alternative pathway, such as the glycine cleavage system (SSCH_1050014-19, SSCH_1050027, SSCH_290017, SSCH_240016), and serine ammonium lyase (SSCH_2660002), were not expressed (Fig 4). It still needs to be investigated whether the prevailing acetate concentration has a regulatory impact on the SAO pathway expressed. Genes of the methyl branch are separately located in the genome (FTHFS, product of SSCH_370012; methylenetetrahydrofolate dehydrogenase/methenyltetrahydrofolate cyclohydrolase, product of SSCH_630016, SSCH_630017) and could therefore be employed by the alternative route when substrate limitation occurs. However, the formation of serine from pyruvate by the activity of serine ammonium lyase is highly endergonic (+44 kJ/mol), what makes an involvement of this enzyme in the assumed direction questionable.

### Energy-conservation during acetate oxidation

*S*. *schinkii* appears to be very well equipped with energy-conserving systems, including e.g. Rnf complex and an Ech hydrogenase (Fig 2, S6 Table).

**Fig 5:**
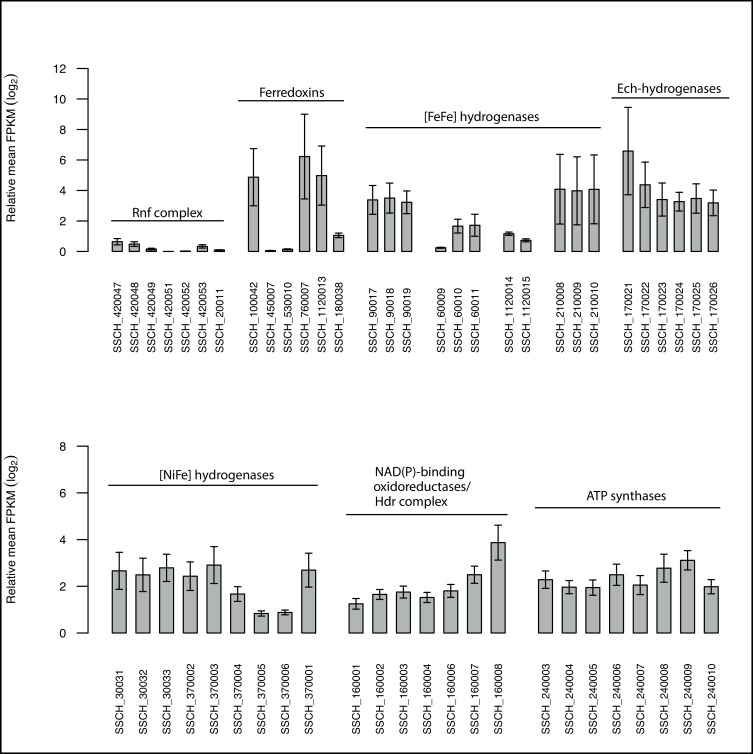
Bar graph showing the FPKM values of genes related to energy conservation.

The six subunits of the respiratory Rnf complex are encoded by the putative operon *rnfCDGEAB* (SSCH_420047–420053), which utilises the redox span between ferredoxin (E^0^`= -400 mV) and NADH (E^0^`= -320 mV) to form an ion gradient [76]. RnfA, D, and E were predicted as integral membrane proteins and subunits C and B have two ferredoxin domains with [4Fe-4S] clusters. The single steps are mechanistically reversible. In *Clostridium kluyveri*, *A*. *woodii* and *C*. *ljungdahlii*, the Rnf complex has been shown to play an important role in energy metabolism by coupling electron flow from reduced ferredoxin to NAD^+^ to proton translocation [77–79]. Interestingly, the Rnf complex appears to have no such role in energy conservation in *S*. *schinkii*, as the transcription level was very low under acetate oxidizing conditions (Fig 5). This agrees with the lack of Rnf-related genes in the genome of the closest relative, the thermophilic acetate oxidizing *T*. *phaeum* [40].

**Fig 6:**
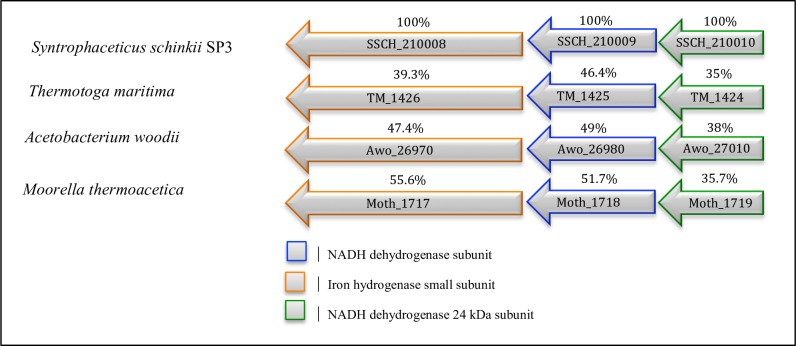
Comparison of the NADH-dependent [Fe-Fe] hydrogenase gene cluster (SSCH_210008–10) predicted for *S*. *schinkii* strain Sp3 to the characterised electron-bifurcating NADH ferredoxin-dependent [Fe-Fe] hydrogenase gene cluster found in *T*. *maritima* and the model acetogens *M*. *thermoacetica* and *A*. *woodii*. Percentage identity numbers of amino acid sequence are given.

It is striking to note the high number of hydrogenases encoded by the genome. A total of four potential [Fe-Fe] hydrogenase gene clusters (SSCH_90017–19, SSCH_60009–11, SSCH_1120014–15, SSCH_210008–10; Fig 6 and S5 Fig, S6 Table) were predicted (see reference [80] for classification of hydrogenases). Cluster SSCH_21008–10 includes genes homolog to the characterised electron-bifurcating ferredoxin- and NAD+-dependent [Fe-Fe] hydrogenases gene clusters of *M*. *thermoacetica*, *A*. *woodii* and *Thermotoga maritima* [81, 82] with the same synteny as found in *S*. *schinkii* (Fig 6). This hydrogenase couples the favourable H_2_ production from reduced ferredoxin to the less favourable H_2_ production from NADH. Several ferredoxin-encoding genes were found dispersed in the genome of *S*. *schinkii* (SSCH _100042, SSCH_450007, SSCH_530010, SSCH_760007, SSCH_1120013) and one putative rubredoxin gene (SSCH_180038).

Potential bifurcating hydrogenases have also been predicted for *S*. *wolfei*, another syntrophic metaboliser producing high molar ratios of H_2_ [83], and for the SAOB *T*. *acetatoxydans* [42] and *T*. *phaeum* [40]. It has been assumed for *T*. *phaeum* that the bifurcating hydrogenase can connect, directly or indirectly via menaquinone to the oxidation of methylene tetrahydrofolate. For *S*. *schinkii*, the transcriptome revealed that two of the [FeFe] hydrogenases, including the predicted bifurcating [FeFe] hydrogenase, and three of the ferredoxins were expressed under acetate oxidising conditions (Fig 5). Therefore, a potential proton motive force could be generated by cytoplasmatic proton consumption.

In addition, the genome encodes an energy-conserving hydrogenase (Ech), a membrane-integral [Ni-Fe] hydrogenases, with the same synteny as found and described for *M*. *barkeri* [84], the sulphate reducer *Desulfovibrio gigas* [85], and the thermophilic SAOB *T*. *phaeum* [40] (S6 Table, S5 Fig), and which appears to be of importance for energy conservation in *S*. *schinkii* as all subunits were expressed under syntrophic growth conditions (Fig 5). The Ech hydrogenase might contribute to the proton motive force by coupling proton translocation across the membrane to the oxidation of reduced ferredoxin and H_2_ formation [84], forming a proton motive force. The ATP synthase operon (SSCH_240003–240010), which is needed for converting the electrochemical gradient into ATP, was expressed (Fig 5).

Another cluster was predicted to encode genes for a periplasmic [NiSeFe] hydrogenase (S6 Table), which is usually associated with H_2_ oxidation and potentially allows the cells to link H_2_ oxidation to anaerobic respiration using CO_2_ as the electron acceptor [86]. It consists of a small subunit (SSCH_30031), a large subunit (SSCH_33032) and a third cytochrome b-like subunit (SSCH_33033). The N-terminus of the small subunit contains a twin arginine motive recognised by the twin-arginine translocation (TAT) translocase (SSCH_170020, SSCH_360036). The cluster showed synteny to a [NiSeFe] hydrogenase cluster found in *T*. *phaeum*, in *Carboxidothermus hydrogenoformans* and in *Desulfosporosinus orientis*, with descending similarity (S6 Fig). The maturation proteins (SSCH_60028–30) are encoded elsewhere in the genome. In the sulphate reducer *Desulfovibrio vulgaris* the expression of the [NiFeSe] hydrogenase is strongly associated with the oxidation of H_2_ [87]. In case of *S*. *schinkii* the transcriptome revealed expression under H_2_ producing conditions (Fig 5). A second putative [NiFe] hydrogenase is very likely cytoplasmic, since it lacks any signal peptides (SSCH_370002–6) and which was likewise expressed (Fig 5, S6 Table). Representatives of this [Ni-Fe] hydrogenase group are reported to function as intracellular H_2_ sensors triggering reaction cascades connected to energy-transducing reactions [86]. The presence of an adjacent predicted response regulator receiver gene (SSCH_370001) might point to a similar function in *S*. *schinkii*.

The genome further encodes a NAD(P)-binding oxidoreductase/heterodisulphide reductase complex (SSCH_160001–8; S7 Fig, S6 Table), which is in synteny to that found in *S*. *wolfei* [83] and in other syntrophic bacteria such as *Syntrophorhabdus aromaticivorans* [88, 89]. It consists of the heterodisulphide reductase subunits A, B and C, three Fe-S proteins and a NAD(P) binding oxidoreductase, and is postulated to be involved in reverse electron transport [88]. The redox pair remains unknown. The presence of Rnf complex and Fd:NADH oxidoreductase/heterodisulphide reductase encoding genes within the same genome appears to be unique to *S*. *schinkii*, since this combination has been reported to be untypical for organisms capable of syntrophic metabolism [43]. However, as described above the Rnf complex does not seem to be of importance for energy conservation of this organism when oxidising acetate, whereas the latter might do as indicated by the transcriptome (Fig 5).

### Acetate uptake and activation

**Fig 7:**
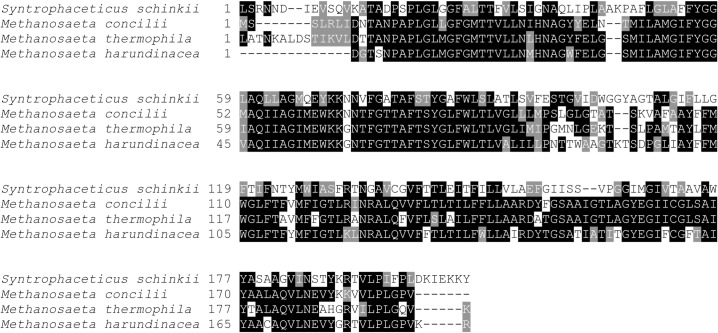
Multiple sequence alignment of the deduced amino acid sequences of the putative acetate transporter predicted for *Syntrophaceticus schinkii* and three *Methanosaeta* genomes.

*Syntrophaceticus schinkii* has been found at high abundance in both low- and high-ammonia conditions, suggesting that this species has a strong competitive ability [15, 25, 46, 60]. However, the poor metabolic capacities uncovered here and the slow heterotrophic growth rates demonstrated cannot explain its competitiveness in biogas processes. The genome harbours an ORF (SSCH_800020), predicted to encode a transporter, which were found expressed in the syntrophic co-cultures (Fig 1). It shows 35–41% identity to a potential acetate transporter predicted for three *Methanosaeta* genomes (Fig 7, S2 Table) [90].

Within the methanogenic Archaea, *Methanosaeta* species can utilise acetate concentrations from as low as 7 to 70 μM. In contrast, *Methanosarcina* species have a minimum acetate concentration threshold between 0.2 to 1.2 mM. Thus, *S*. *schinkii* might have the ability to compete for acetate with *Methanosaeta* species, which dominate the methanogenic community at low ammonia levels, and *Methanosarcina* species, which are prominent under SAO conditions [19], and might exclusively rely on the continuously produced key intermediate acetate.

Under non-acetate limiting conditions as applied in the present study, acetate appears to be activated by the activities of phosphotransacetylase and acetate kinase, of which both were found expressed (Fig 6), consuming one ATP. However, *S*. *schinkii* might increase its competitiveness by employing an archaeal-like ADP-forming acetyl-CoA synthase (product of SSCH_480004; S8 Fig) for acetate activation, when acetate concentration becomes crucial. The archaeal ADP-forming acetyl-CoA synthase has a much lower k_m_ for acetate (340–660 μM) [91–93] than the archaeal or bacterial acetate kinases (7–22 mM) [94, 95].

Moreover, *S*. *schinkii* harbours three genes (SSCH_1190010, SSCH_330008, SSCH_1000017; S2 Table) predicted to belong to the sodium:solute symporter (TC 2.A.21) family and sharing 24, 24 and 22% identity with MctC, MctP and ActP, respectively. These genes have been identified as acetate transporters in *Corynebacterium glutamicum* [96], *Rhizobium leguminosarum* [97] and *E*. *coli* [98], respectively. A transporter belonging to the same family has recently been predicted for the SAOB *T*. *acetatoxydans* [42]. In addition, *S*. *schinkii* contains a gene encoding a potential formate/nitrite transporter (FNT, product of SSCH_150019; S2 Table), similar to that predicted in *T*. *acetatoxydans*, which might play a role in acetate transport [42]. However, none of these gene products was expressed under the growth conditions investigated (Fig 1).

## Conclusions

The lack of flagella, chemotactic behaviour and limited metabolic capacities imply inability of *S*. *schinkii* to adapt to rapidly changing conditions. This can be considered an adaptation to the AD environment, which is nutrient-rich and where precursors become continuously available. Based on the genomic traits predicted, it is likely that *S*. *schinkii* cells employ type IV pili and quorum sensing for synchronising activities and communication with the methanogenic partner, in order to initiate and stabilise intimate syntrophy, a prerequisite for occupying a similar niche as the non-syntrophically living aceticlastic methanogens. Natural and artificial carriers might be supportive for establishing SAO, since *S*. *schinkii* appears to be motile through gliding. Furthermore, surface attachment attributes reduce the risk of washout during process operation, while quorum sensing maintains communication.

*S*. *schinkii* is a highly specialised, habitat-adapted organism. It appears to be on the verge of being an obligate syntrophic organism, which relies on SAO rather than on metabolic versatility, occupying a similar niche as the aceticlastic methanogens. By expanding its complement of respiratory protein complexes, it overcomes limiting bioenergetics barriers, enabling efficient energy conservation from reactions operating close to thermodynamic equilibrium and driving thermodynamically unfavourable reactions. *Syntrophaceticus schinkii* has great potential to serve as a model organism for studying syntrophic relationships and SAO-related issues in future -omics approaches aiming to specify process conditions supporting efficient and robust bio-hydrogen and bio-methane production.

## Supporting Information

S1 Appendix ClustalW alignment file of the deduced ADP-forming acetyl-CoA synthase including the closest 100 hits obtained by the BLASTP search algorithm using default parameters. (TXT)

S2 Appendix Cofactor biosynthesis. (DOCX)

S3 Appendix General genome features. (DOCX)

S1 Fig Bar graph showing the FPKM values of genes related to type IV pili and quorum sensing (QS). (PDF)

S2 Fig Bar graph showing the FPKM values of genes related to heat shock proteins. (PDF)

S3 Fig Bar graph showing the FPKM values of genes related to heterotrophic metabolism and gluconeogenesis. Sel, selenocysteine-decoding machinery; ADH, alcohol dehydrogenase; PS, pyruvate synthase; PFL, pyruvate formate lyase; TMA, trimethylamine metabolism; EMP, Embden-Meyerhof-Parnas pathway.
(PDF)

S4 Fig Comparison of the structures of Wood-Ljungdahl pathway gene clusters found in the SAOB *Syntrophaceticus schinkii*, *Thermacetogenium phaeum* and the acetogens *Moorella thermoacetica*, *Acetobacterium woodii* and *Clostridium ljungdahlii*. (PDF)

S5 Fig Comparison of the NADH-dependent [Fe-Fe] hydrogenase and the energy-conserving hydrogenase (Ech) hydrogenase gene cluster. A) Comparison of the NADH-dependent [Fe-Fe] hydrogenase gene cluster (SSCH_600009–11, 90017–19, 1120014–15, 210008–10) predicted for *Syntrophaceticus schinkii* strain Sp3 to NADH ferredoxin-dependent [Fe-Fe] hydrogenase gene clusters found in *Thermotoga maritima* and the acetogens *Moorella thermoacetica* and *Acetobacterium woodii*. B) Comparison of the energy-conserving hydrogenase (Ech) hydrogenase gene cluster predicted in *Syntrophaceticus schinkii* strain Sp3 to the Ech hydrogenase clusters found in the SAOB *Thermacetogenium phaeum*, the sulphate reducer *D*. *giga*s and the methanogen *Methanosarcina barkeri*. Percentage identity numbers of amino acid sequence are given.
(PDF)

S6 Fig Comparison of the periplasmic [Ni-Fe] hydrogenase gene cluster. Comparison of the periplasmic [Ni-Fe] hydrogenase gene cluster predicted for *S*. *schinkii* strain Sp3 to the [Ni-Fe] hydrogenase gene clusters found in the genome of the SAOB *T*. *phaeum*, the hydrogen-producing *Carboxidothermos hydrogenoformans* and the sulphate reducer *Desulfosporosinus orientis*. Percentage identity numbers of amino acid sequence are given.
(PDF)

S7 Fig Comparison of the putative Ferredoxin:NADH oxidoreductase/heterodisulphide reductase complex gene cluster to a similar gene cluster found in *S*. *wolfei*. *Syntrophorhabdus aromaticivorans* could not be included in the comparison since the gene sequences of the locus tags published are not publicly available. Percentage identity numbers of amino acid sequence are given.
(PDF)

S8 Fig Multiple sequence alignment of the deduced amino acid sequences of the putative archaeal-like ADP-forming acetyl-CoA synthase of *S*. *schinkii* and ADP-forming acetyl-CoA synthases of selected members of the domain Archaea. A comprehensive alignment file can be found in Additional file 15.
(PNG)

S1 Table Gene loci in *Syntrophaceticus schinkii* predicted to encode type IV pilus-related proteins, sporulation related proteins, and pyrrolysine biosynthesis related proteins. (DOC)

S2 Table Transporters predicted for the genome of *Syntrophaceticus schinkii*. (DOCX)

S3 Table Genes predicted in *Syntrophaceticus schinkii* to be related to trimethylamine metabolism. (DOC)

S4 Table Gene clusters in *Syntrophaceticus schinkii* predicted to encode the Embden-Meyerhof-Parnas pathway. (DOC)

S5 Table Genes associated to functions in the Wood-Ljungdahl pathway in *Syntrophaceticus schinkii*. (DOCX)

S6 Table Genes potentially involved in electron transfer mechanisms in *Syntrophaceticus schinkii*. (DOC)
