# Supplementary material for: Genome-Guided Analysis and Whole Transcriptome Profiling of the Mesophilic Syntrophic Acetate Oxidising Bacterium Syntrophaceticus schinkii
Source: PLoS One. 2016 Nov 16;11(11):e0166520. doi: 10.1371/journal.pone.0166520 (PMC5113046; doi:10.1371/journal.pone.0166520)
Supplement: S2 Appendix — (DOCX) [file pone.0166520.s002.docx]

**Cofactor biosynthesis**

Laboratory cultivation of *S. schinkii* requires a high level of nutrient supply. This can probably be explained by the fact that the genome lacks the genes needed for *de novo* synthesis of coenzyme precursors, such as nicotinic acid, folic acid, pyridoxine, thiamine, biotin and pantothenic acid derivatives, possibly as an adaptation to nutrient-rich environments. Like *T. acetatoxydans*, *S. schinkii* has been exclusively detected in AD processes (comparison of the 16S rRNA gene sequence with the latest available databases from GenBank (2015-08-11) using NCBI BLASTN under default settings).

In both the WL pathway and the alternative pathway described, tetrahydrofolate is one of the important cofactors. Two metabolic routes for the synthesis of tetrahydrofolate are the *de novo* synthesis pathway and a salvage pathway. Similarly to *T. phaeum*, for the *de novo* synthesis pathway all genes required (folE: SSCH_600015, folB:SSCH_370015, folk:SSCH_370016, folP:SSCH_370014, folC:SSCH_450009) to produce 6-(S)-tetrahydrofolate are present except one, the dihydrofolate reductase gene (folA). However, an alternative enzyme, dihydropteridine reductase, also encoded in *S. schinkii* (SSCH_1160025), has been suggested to fulfil this role in *T. phaeum* [1]. The functions of the tetrahydrofolate salvage pathway in *S. schinkii*, which regenerates tetrahydrofolate from 5- or 10-formyl-tetrahydrofolate, could be accomplished by methylenetetrahydrofolate dehydrogenase/methenyltetrahydrofolate cyclohydrolase of the WL pathway (SSCH_630016-630017 and SSCH_1250009) reactions, although requiring folic acid as a growth supplement.

Another necessary cofactor in the Wood-Ljungdahl pathway is cobalamin, which is involved in the activation of several methylated compounds. Both the *T. phaeum* and *S. schinkii* genome lack essential genes of the anaerobic ‘early’ cobalt insertion pathway, for which products such as a chelatase complex are needed for cobalt insertion as the central atom of cobalamine. It has been suggested [1], that those missing functions might be replaced by other gene products. 5-aminolevulinate, a precursor for tetrapyrroles, can be formed by glutamyl-tRNA synthase (product of SSCH_570013), glutamyl-tRNA reductase (product of SSCH_360038) and glutamate-1-semialdehyde 2,1-aminomutase (product of SSCH_600048/49). Moreover, the activities of porphobiligogen synthase (product of SSCH_600053), porphobiligogen deaminase (product of SSCH_360039) and uroporphyrinogen III synthase (product of SSCH_600056) can produce uroporphyrinogen III, a precursor for biosynthesis of different tetrapyrrole compounds, such as cobalamine and haem. All genes necessary for protohaem synthesis from uroporphyrinogen III were also found (SSCH_270008, SSCH_170007, SSCH_360037-38) except for the protoporphyrinogen IX dehydrogenase. Since a subunit of both a formate dehydrogenase and a [Ni-Fe] hydrogenase were predicted to carry a haem b cofactor, *S. schinkii* might possess mechanisms for haem and Fe acquisition from the environment. None of the genes was found expressed at higher levels, but the co-cultures were supplemented with any precursor needed for growth (data not shown).

References

1. Oehler D, Poehlein A, Leimbach A, Muller N, Daniel R, Gottschalk G, et al. Genome-guided analysis of physiological and morphological traits of the fermentative acetate oxidizer Thermacetogenium phaeum. BMC Genomics 2012;13:723.
