# Supplementary material for: Genome-Guided Analysis and Whole Transcriptome Profiling of the Mesophilic Syntrophic Acetate Oxidising Bacterium Syntrophaceticus schinkii
Source: PLoS One. 2016 Nov 16;11(11):e0166520. doi: 10.1371/journal.pone.0166520 (PMC5113046; doi:10.1371/journal.pone.0166520)
Supplement: S3 Appendix — (DOCX) [file pone.0166520.s003.docx]

**General genomic features**

The major general genomic features of *Syntrophaceticus schinkii* strain Sp3 are presented in [1]. The total size of the working draft genome sequence of *Syntrophaceticus schinkii* strain Sp3 used in this study was 3,166,921 bp and is publicly available at <http://www.ebi.ac.uk/ena/data/view/PRJEB5769>.

The closest current relative of the mesophilic *S. schinkii* is the thermophilic SAOB *T. phaeum*, showing 92.12% 16S rRNA gene identity (NCBI BLASTN search 2015-08-30). Synteny analysis with all genomes available in the Prokaryotic Genome Data Base (PkGDB; 2015-08-30) also showed the maximum number of orthologues (1,788 or 51.9%) to *T. phaeum*. The genome contains 2,586 predicted protein-coding sequences (CDS), of which 75.05% can be allocated to the 21 functional Cluster of Orthologous Groups (COGs), a similar range as found for *T. phaeum* [2]. The largest numbers of genes fall into four main categories: amino acid transport and metabolism (9.84%), energy production and conservation (5.92%), inorganic ion transport and metabolism (5.97) and carbohydrate transport and metabolism (4.00). Similar numbers of tandem duplicated protein-coding genes are present in the genome of *S. schinkii* (163 duplicated genes) and *T. phaeum* (176 duplicated genes). Tandem duplications may facilitate adaptation by the organisms to diverse conditions in their respective niche and may augment protein expression, allowing the cells to proliferate under growth-limiting and competitive conditions [3,4]. The genome of *S. schinkii* shows indications that this organism might have to cope with mobile genetic elements in its environment, as reflected by the high number of Clustered Regularly Interspaced Short Palindromic Repeats (CRISPR) loci found (Table 1). The cas/CRISPR system is widespread in many bacterial and almost all archaeal genomes sequenced [5], and is considered a prokaryotic defence mechanism that provides immunity against invading mobile genetic elements such as phages and plasmids in an RNA interference-like manner [6].

Table 1: CRISP (Clustered Regularly Interspaced Short Palindromic Repeats) loci predicted in the genome of *Syntrophaceticus schinkii* strain Sp3

| **Locus Tag** | | **Begin** | **End** | **Length** | **Gene** | **Product** |
| --- | --- | --- | --- | --- | --- | --- |
| **CRISPR loci** | | | | | | |
| SSCH_30001 | 50519 | | 51181 | 663 | *_* | CRISPR-associated protein TM1801 |
| SSCH_30003 | 51324 | | 51824 | 501 | *_* | CRISPR-associated endonuclease Cas3-HD |
| SSCH_30004 | 51831 | | 53480 | 1650 | *_* | CRISPR-associated HD domain-containing protein |
| SSCH_830002 | 2036390 | | 2036692 | 303 | *cas2* | CRISPR-associated endoribonuclease Cas2 |
| SSCH_830003 | 2036703 | | 2036882 | 180 | *_* | CRISPR-associated endonuclease Cas1 1 |
| SSCH_830004 | 2036743 | | 2037735 | 993 | *cas1* | CRISPR-associated endonuclease Cas1 1 |
| SSCH_830005 | 2037732 | | 2038427 | 696 | *cas4* | CRISPR-associated exonuclease, Cas4 family |
| SSCH_830006 | 2038429 | | 2039514 | 1086 | *_* | CRISPR-associated protein, Csd2 family |
| SSCH_830007 | 2039552 | | 2041294 | 1743 | *_* | Csd1 family CRISPR-associated protein |
| SSCH_830008 | 2041291 | | 2042022 | 732 | *cas5* | CRISPR-associated protein Cas5 |
| SSCH_830009 | 2042040 | | 2043749 | 1710 | *cas3* | CRISPR-associated nuclease/helicase Cas3 |
| SSCH_1990003 | 2996122 | | 2996724 | 603 | *cas5* | CRISPR-associated protein Cas5 |
| SSCH_2140001 | 3035736 | | 3036197 | 462 | *_* | CRISPR-associated helicase Cas3 |

Only a small number of organisms [7], including the SAOB *T. acetatoxydans* [8] and *T. phaeum* [2], have been found to harbour 10 or more CRISPRs loci. The genome of *S. schinkii* contains eight CRISPR loci (Table S1) and one operon encoding the CRISPR-associated sequence (*cas*) genes (SSCH_830002-830009). We also found one region in the genome of *S. schinkii* that harbours prophage-related genes. Similarly to *S. schinkii*, the genome of both *T. phaeum* and *T. acetatoxydans* have been shown to harbour prophages and prophage-related genes [2,8]. The acquisition of numerous CRISPR loci observed in all three SAOB genomes can most likely be considered an adaptation to the habitat, which might be continuously subjected to phage attacks, as previously suggested by Müller et al. [8].

References

1. Manzoor S, Müller B, Niazi A, Schnürer A, Bongcam-Rudloff E. Working draft genome sequence of the mesophilic acetate oxidizing bacterium Syntrophaceticus schinkii strain Sp3. Stand Genomic Sci. 2015; doi: 10.1186/s40793-015-00922015.

2. Oehler D, Poehlein A, Leimbach A, Muller N, Daniel R, Gottschalk G, et al. Genome-guided analysis of physiological and morphological traits of the fermentative acetate oxidizer Thermacetogenium phaeum. BMC Genomics 2012;13:723.

3. Reams AB, Neidle EL. Selection for gene clustering by tandem duplication. Annu Rev Microbiol. 2004;58:119–42.

4. Romero D, Palacios R. Gene amplification and genomic plasticity in prokaryotes. Annu Rev Genet. 1997;31:91–111.

5. Horvath P, Barrangou R. CRISPR/Cas, the immune system of bacteria and archaea. Science 2010;327:167–70.

6. Barrangou R, Fremaux C, Deveau H, Richards M, Boyaval P, Moineau S, et al. CRISPR provides acquired resistance against viruses in prokaryotes. Science 2007;315:1709–12.

7. Manzoor S. Computational and comparative Investigations of syntrophic acetate-oxidosing bacteria (SAOB) - Genome-guided analysis of metabolic capacities and energy conserving systems. Swedish Universtiy of Agricultural Sciences. Microbiology. 2014; Doctoral Thesis No. 2014:56

8. Müller B, Manzoor S, Niazi A, Bongcam-Rudloff E, Schnürer A. Genome-Guided

Analysis of Physiological Capacities of Tepidanaerobacter acetatoxydans Provides

Insights into Environmental Adaptations and Syntrophic Acetate Oxidation. PLoS

One. 2015;10:DOI: 10.1371/journal.pone.0121237
