# Supplementary figures and images for: Genome-Guided Analysis and Whole Transcriptome Profiling of the Mesophilic Syntrophic Acetate Oxidising Bacterium Syntrophaceticus schinkii
Source: PLoS One. 2016 Nov 16;11(11):e0166520. doi: 10.1371/journal.pone.0166520 (PMC5113046; doi:10.1371/journal.pone.0166520)

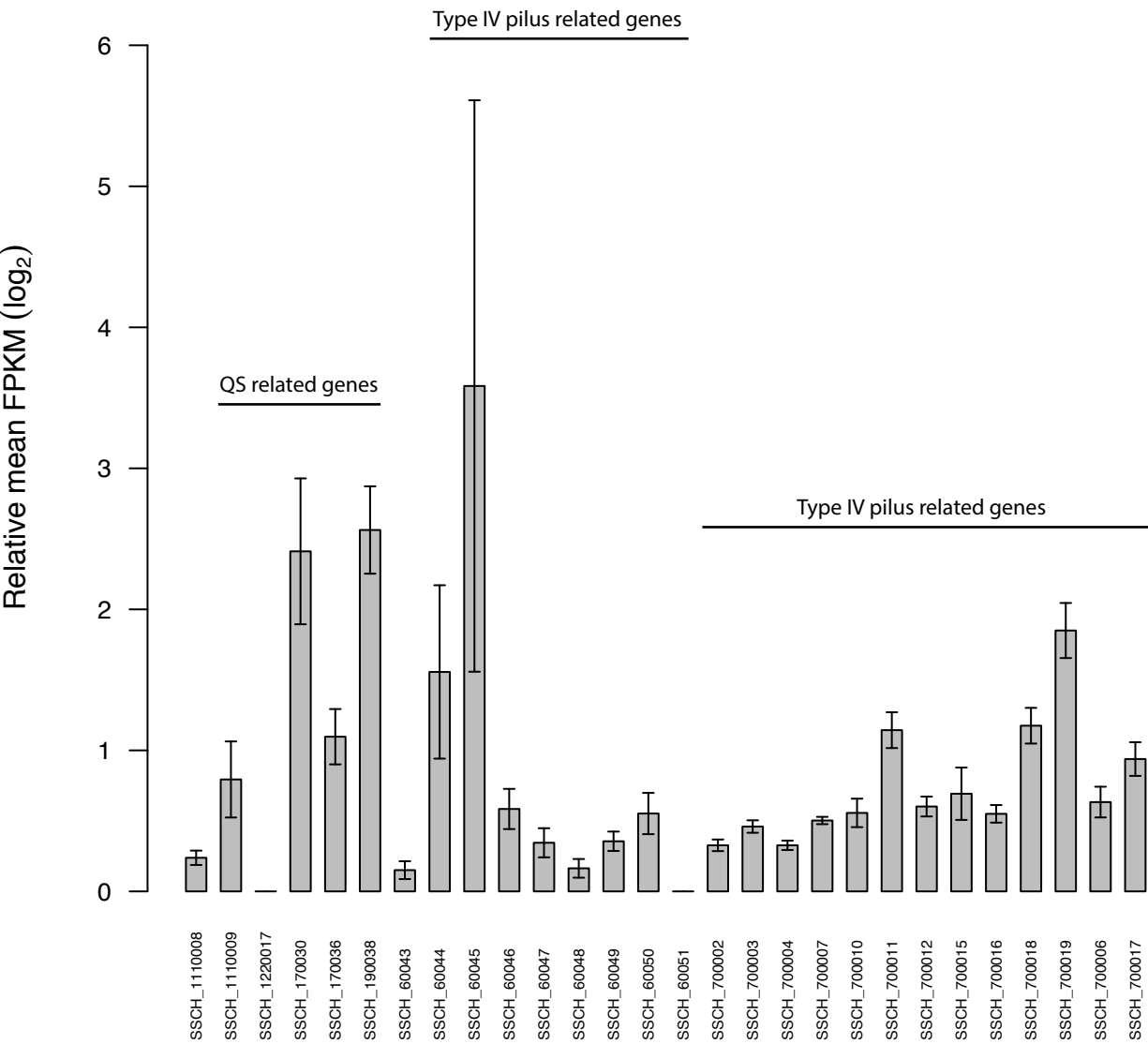

Supplement: S1 Fig — (PDF) [file pone.0166520.s004.pdf]

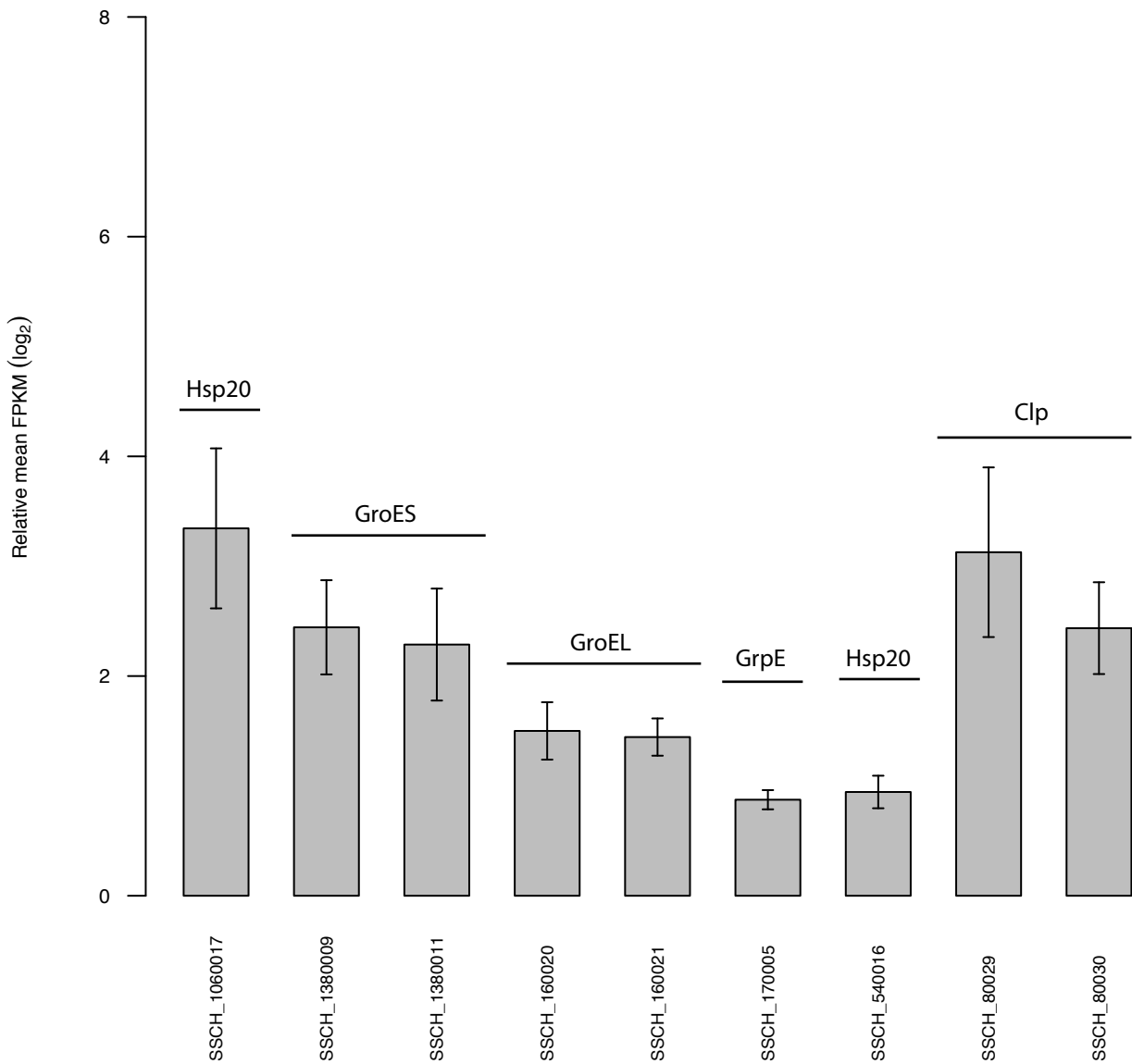

Supplement: S2 Fig — (PDF) [file pone.0166520.s005.pdf]

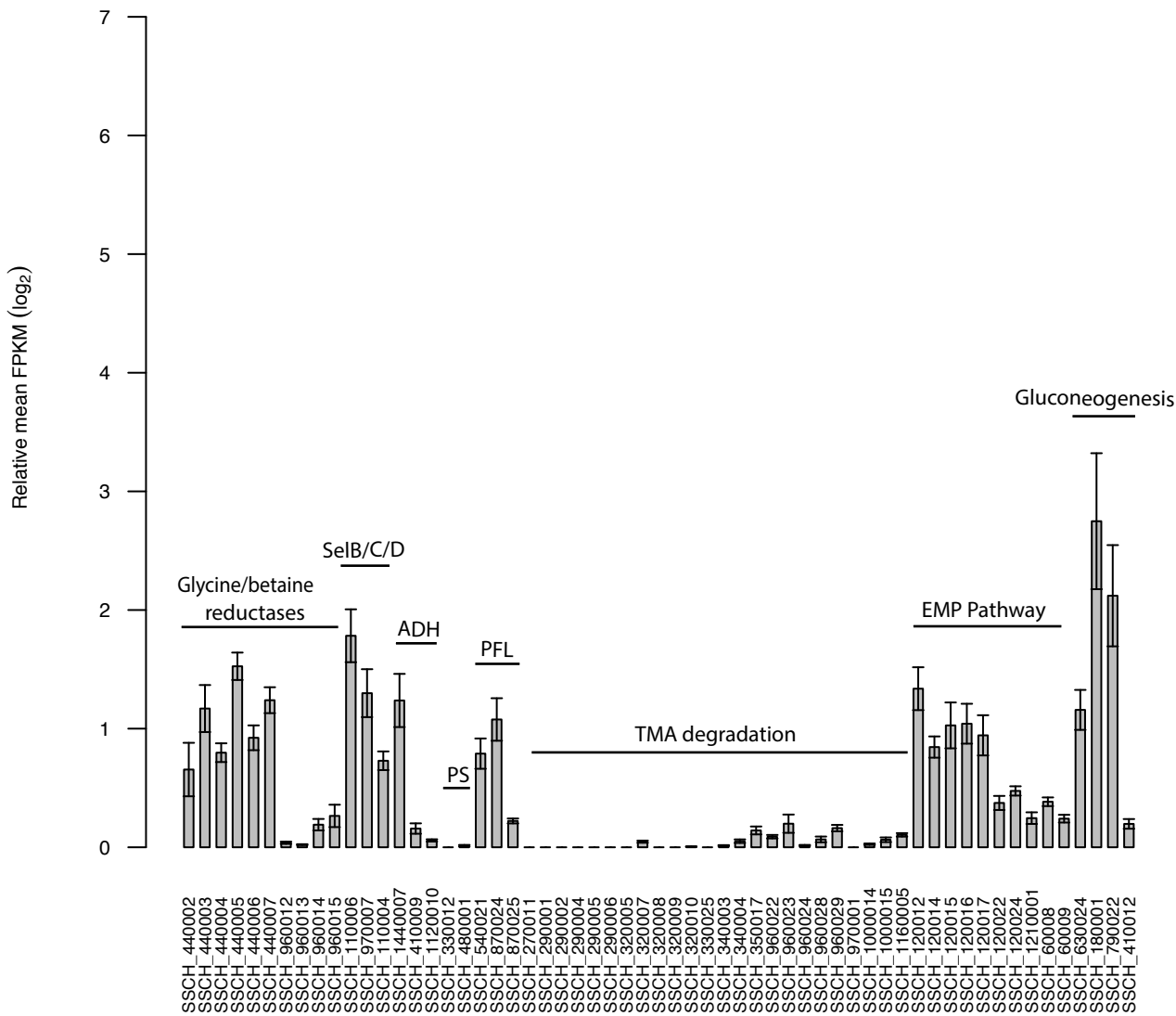

Supplement: S3 Fig — Sel, selenocysteine-decoding machinery; ADH, alcohol dehydrogenase; PS, pyruvate synthase; PFL, pyruvate formate lyase; TMA, trimethylamine metabolism; EMP, Embden-Meyerhof-Parnas pathway. (PDF) [file pone.0166520.s006.pdf]

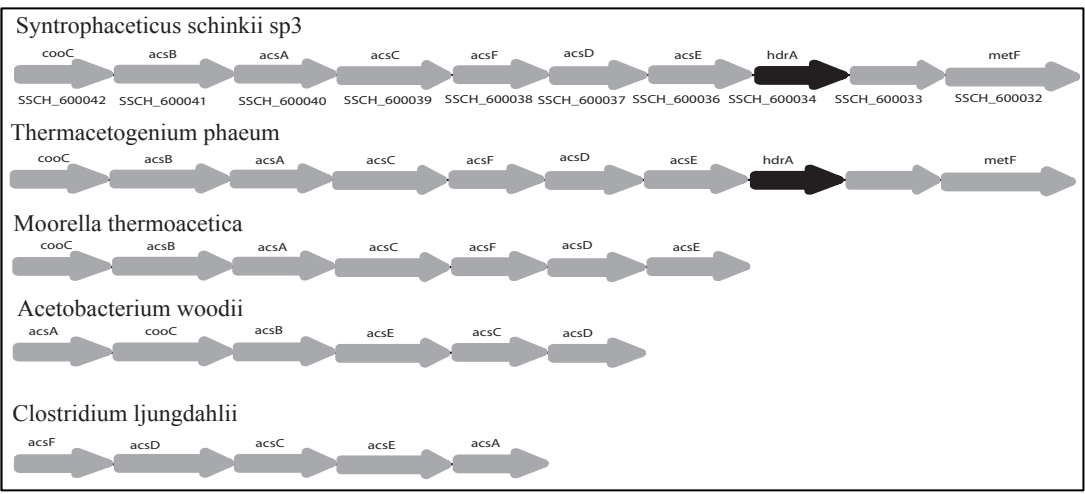

Supplement: S4 Fig — (PDF) [file pone.0166520.s007.pdf]

A.

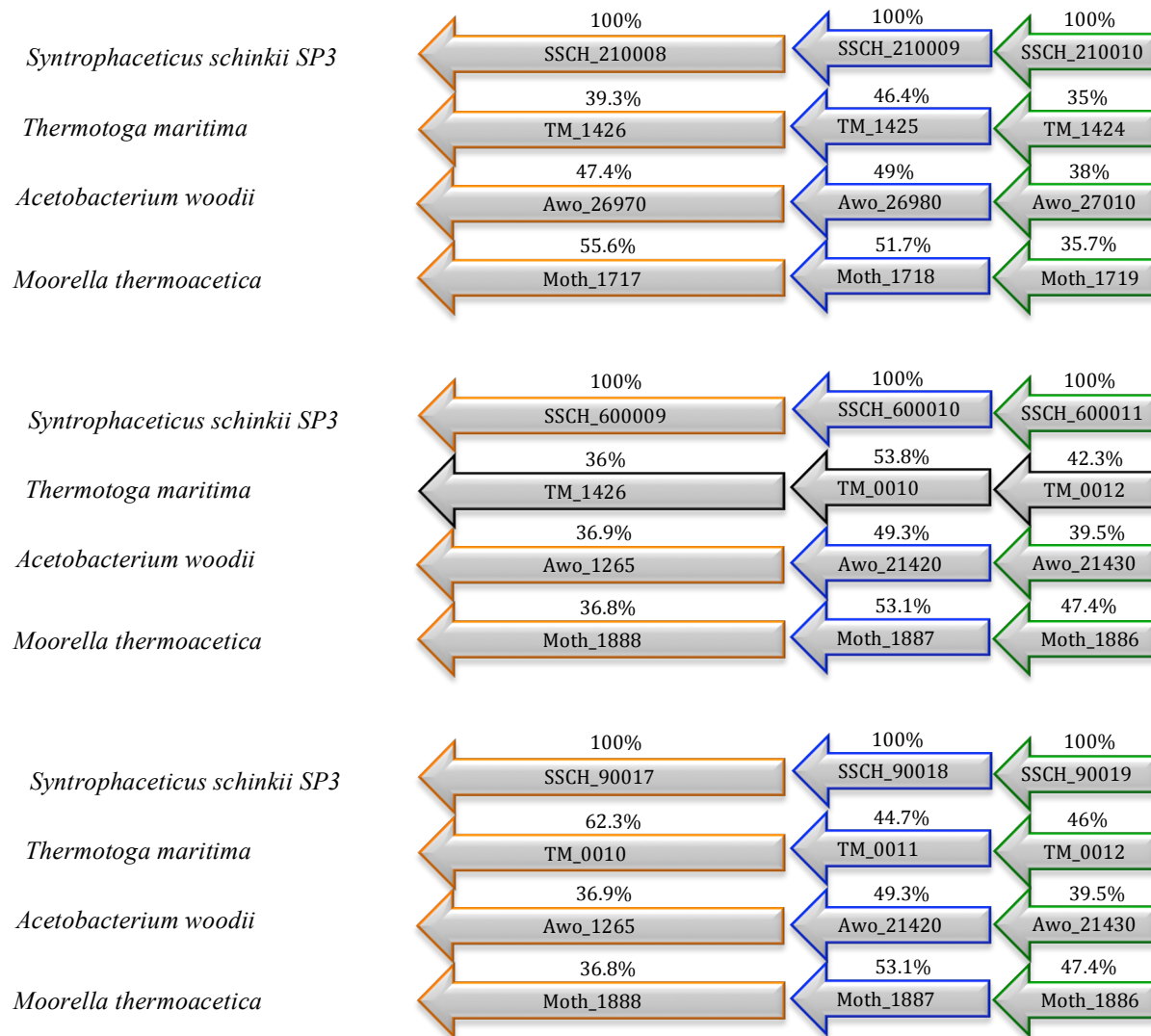

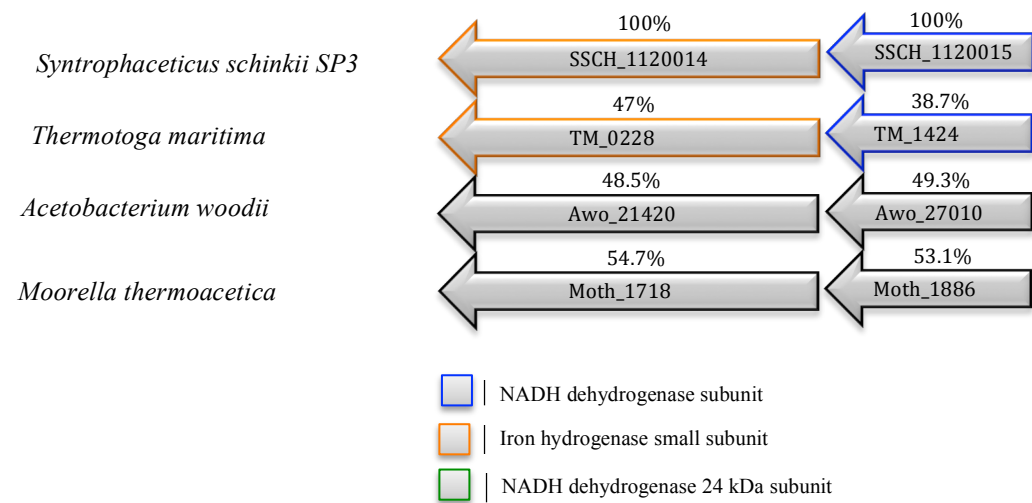

B.

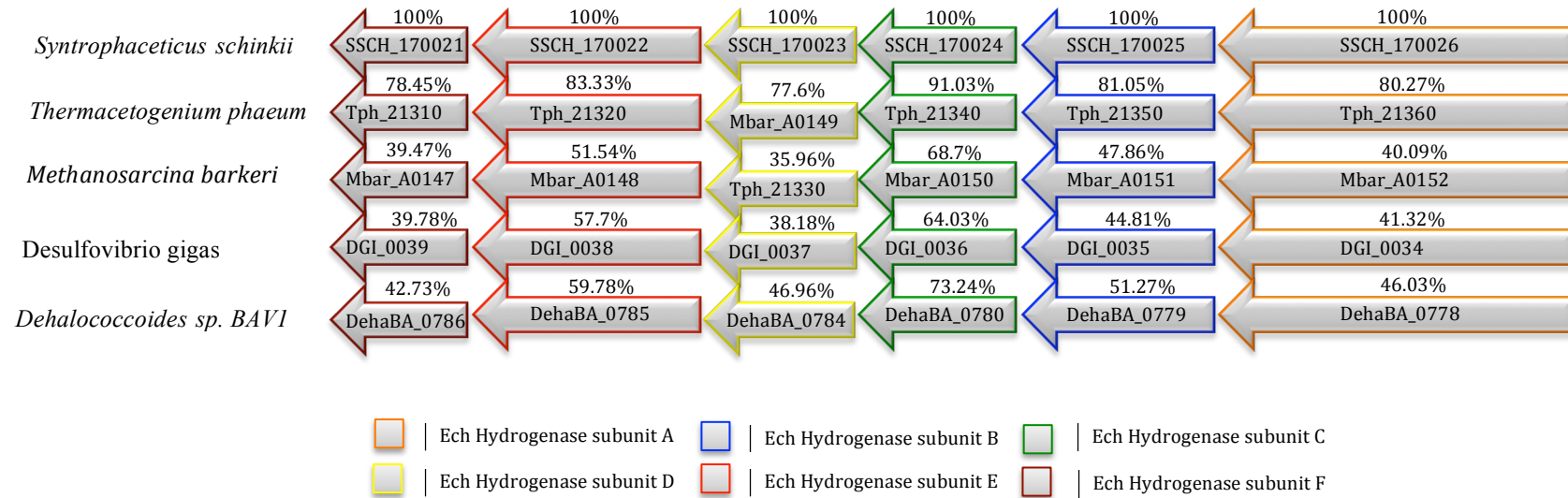

Supplement: S5 Fig — A) Comparison of the NADH-dependent [Fe-Fe] hydrogenase gene cluster (SSCH_600009–11, 90017–19, 1120014–15, 210008–10) predicted for Syntrophaceticus schinkii strain Sp3 to NADH ferredoxin-dependent [Fe-Fe] hydrogenase gene clusters found in Thermotoga maritima and the acetogens Moorella thermoacetica and Acetobacterium woodii. B) Comparison of the energy-conserving hydrogenase (Ech) hydrogenase gene cluster predicted in Syntrophaceticus schinkii strain Sp3 to the Ech hydrogenase clusters found in the SAOB Thermacetogenium phaeum, the sulphate reducer D. gigas and the methanogen Methanosarcina barkeri. Percentage identity numbers of amino acid sequence are given. (PDF) [file pone.0166520.s008.pdf]

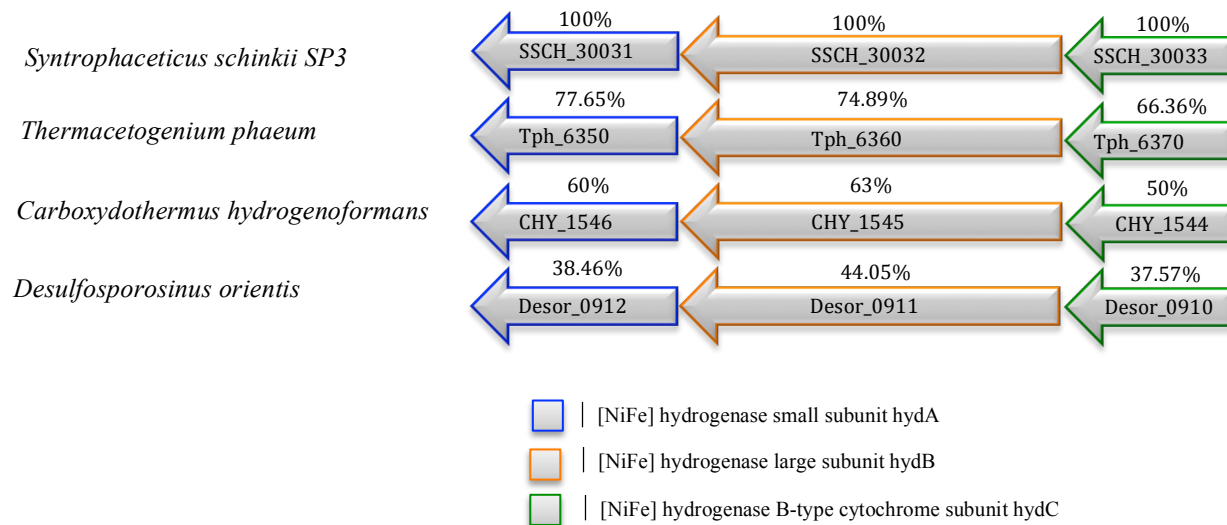

Supplement: S6 Fig — Comparison of the periplasmic [Ni-Fe] hydrogenase gene cluster predicted for S. schinkii strain Sp3 to the [Ni-Fe] hydrogenase gene clusters found in the genome of the SAOB T. phaeum, the hydrogen-producing Carboxidothermos hydrogenoformans and the sulphate reducer Desulfosporosinus orientis. Percentage identity numbers of amino acid sequence are given. (PDF) [file pone.0166520.s009.pdf]

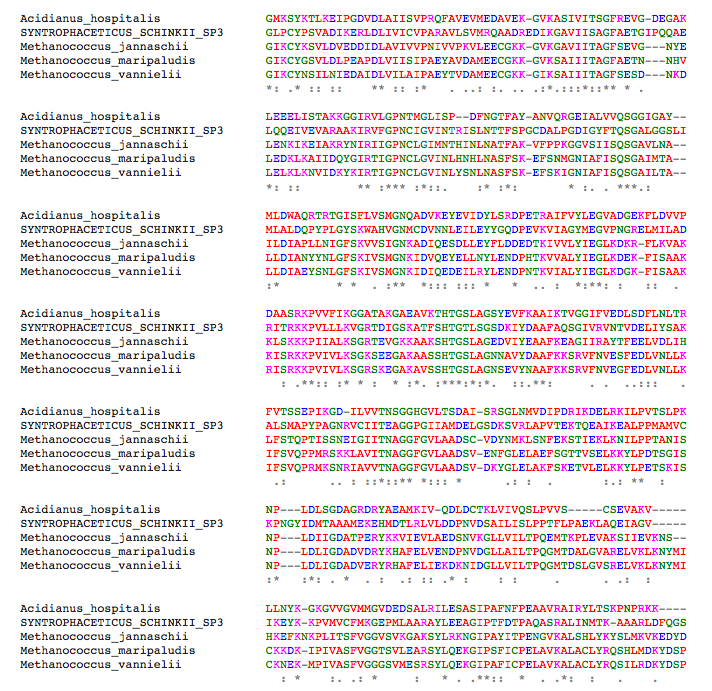

Supplement: S8 Fig — A comprehensive alignment file can be found in Additional file 15. (PNG) [file pone.0166520.s011.png]
