# Supplementary material for: Genome-Guided Analysis and Whole Transcriptome Profiling of the Mesophilic Syntrophic Acetate Oxidising Bacterium Syntrophaceticus schinkii
Source: PLoS One. 2016 Nov 16;11(11):e0166520. doi: 10.1371/journal.pone.0166520 (PMC5113046; doi:10.1371/journal.pone.0166520)
Supplement: S7 Fig — Syntrophorhabdus aromaticivorans could not be included in the comparison since the gene sequences of the locus tags published are not publicly available. Percentage identity numbers of amino acid sequence are given. (PDF) [file pone.0166520.s010.pdf]

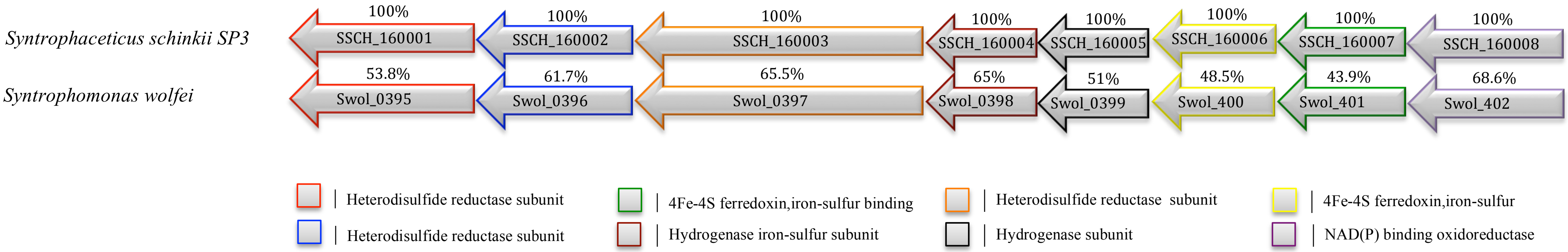
Syntrophaceticus schinkii SP3
Syntrophomonas wolfei
100%
SSCH_160001
100%
SSCH_160002
100%
SSCH_160003
100%
SSCH_160004
100%
SSCH_160005
100%
SSCH_160006
100%
SSCH_160007
100%
SSCH_160008
53.8%
Swol_0395
61.7%
Swol_0396
65.5%
Swol_0397
65%
Swol_0398
51%
Swol_0399
48.5%
Swol_400
43.9%
Swol_401
68.6%
Swol_402
Heterodisulfide reductase subunit
4Fe-4S ferredoxin,iron-sulfur binding
Heterodisulfide reductase subunit
4Fe-4S ferredoxin,iron-sulfur
Heterodisulfide reductase subunit
Hydrogenase iron-sulfur subunit
Hydrogenase subunit
NAD(P) binding oxidoreductase
