# Supplementary material for: Genome-Guided Analysis and Whole Transcriptome Profiling of the Mesophilic Syntrophic Acetate Oxidising Bacterium Syntrophaceticus schinkii
Source: PLoS One. 2016 Nov 16;11(11):e0166520. doi: 10.1371/journal.pone.0166520 (PMC5113046; doi:10.1371/journal.pone.0166520)
Supplement: S1 Table — (DOC) [file pone.0166520.s012.doc]

| **Label** | **Begin** | **End** | **Length (bp)** | **Gene** | **Product** |
| --- | --- | --- | --- | --- | --- |
| SSCH_700002 | 1724070 | 1724924 | 855 | - | Type II secretion system protein |
| SSCH_700003 | 1724943 | 1726046 | 1104 | *pilT* | Twitching motility protein |
| SSCH_700005 | 1726339 | 1726587 | 249 | *pilD* | Type 4 prepilin-like proteins leader peptide-processing enzyme [Includes: Leader peptidase ; N-methyltransferase] |
| SSCH_700006 | 1727266 | 1727766 | 501 | *fimT* | Tfp pilus assembly protein FimT |
| SSCH_700007 | 1727763 | 1728467 | 705 | *pilM1* | Type IV pilus assembly protein PilM |
| SSCH_700008 | 1728469 | 1728624 | 156 | *pilN* | Type IV pilus assembly protein PilN |
| SSCH_700010 | 1728751 | 1729071 | 321 | *-* | Protein of unknown function |
| SSCH_700011 | 1729068 | 1729943 | 876 | *pilM2* | Type IV pilus assembly protein PilM |
| SSCH_700012 | 1730001 | 1731026 | 1026 | *-* | Protein of unknown function DUF583 |
| SSCH_700013 | 1731034 | 1731264 | 231 | *-* | Exported protein of unknown function |
| SSCH_700015 | 1731562 | 1731984 | 423 | *-* | Prepilin-type cleavage/methylation domain containing protein |
| SSCH_700016 | 1731985 | 1732602 | 618 | *-* | Prepilin-type cleavage/methylation domain containing protein |
| SSCH_700017 | 1732592 | 1732993 | 402 | *pilA* | Fimbrial protein PilA |
| SSCH_700018 | 1733081 | 1734289 | 1209 | *pilC* | Type 4 fimbrial assembly protein PilC |
| SSCH_700019 | 1734299 | 1735897 | 1599 | *pilB* | Type IV pilus assembly protein PilB |
| SSCH_60044 | 160678 | 160911 | 234 | *pihO* | Type IV pilus biogenesis protein PihO |
| SSCH_60045 | 161141 | 162319 | 1179 | *pihP* | Type IV pilus minor pilin PihP |
| SSCH_60046 | 162909 | 164459 | 1551 | *pihQ* | Type IV pilus minor pilin PihQ |
| SSCH_60047 | 164639 | 166129 | 1491 | *pihI* | Type IV pilus biogenesis ATPase and membrane protein PihI |
| SSCH_60048 | 166143 | 166358 | 216 | *pihJ* | Type IV pilus biogenesis protein PihJ |
| SSCH_60049 | 166424 | 166717 | 294 | *pihJ* | Type IV pilus biogenesis protein PihJ |
| SSCH_60050 | 166718 | 167833 | 1116 | *pilT* | Twitching motility protein |
| SSCH_10008 | 4588 | 4845 | 258 | *spoVG* | Regulator required for spore cortex synthesis (stage V sporulation) |
| SSCH_10037 | 25697 | 26080 | 384 | *_* | Sporulation-specific protease yabG |
| SSCH_30023 | 66370 | 67596 | 1227 | *_* | Putative Sporulation protein YqfD |
| SSCH_50011 | 99245 | 99709 | 465 | *_* | Stage V sporulation protein AEB (modular protein) |
| **Label** | **Begin** | **End** | **Length (bp)** | **Gene** | **Product** |
| SSCH_50012 | 99727 | 100734 | 1008 | *spoVAD* | Stage V sporulation protein AD |
| SSCH_50013 | 100745 | 101056 | 312 | *_* | Stage V sporulation protein AC |
| SSCH_50017 | 102256 | 102594 | 339 | *_* | RNA polymerase sporulation-specific sigma factor (sigma-G) |
| SSCH_50028 | 109303 | 109740 | 438 | *_* | Stage II sporulation protein M |
| SSCH_90001 | 212710 | 213396 | 687 | *_* | Sporulation protein YunB |
| SSCH_100016 | 264601 | 264867 | 267 | *_* | Sporulation protein, YlmC/YmxH family |
| SSCH_100039 | 284138 | 285238 | 1101 | *_* | Putative Sporulation integral membrane protein YtvI |
| SSCH_110008 | 302963 | 303568 | 606 | *_* | Sporulation protein YyaC |
| SSCH_120019 | 336158 | 337090 | 933 | *whiA* | Putative sporulation transcription regulator WhiA |
| SSCH_190036 | 563782 | 564042 | 261 | *spoVS* | Regulator required for dehydratation of the spore core and assembly of the coat (stage V sporulation) |
| SSCH_360006 | 891414 | 891884 | 471 | *_* | Sporulation lipoprotein, YhcN/YlaJ family |
| SSCH_360009 | 892935 | 893102 | 168 | *_* | Putative stage II sporulation protein P (Part 1) |
| SSCH_360010 | 893122 | 893886 | 765 | *_* | Putative stage II sporulation protein P (Part 2) |
| SSCH_360020 | 899560 | 900684 | 1125 | *spoIVA* | Morphogenetic stage IV sporulation protein (part 1) |
| SSCH_360021 | 900611 | 901042 | 432 | *spoIVA* | Morphogenetic stage IV sporulation protein (part 2) |
| SSCH_460001 | 1160166 | 1160891 | 726 | *sigE* | RNA polymerase sporulation-specific sigma-29 factor (sigma-E) |
| SSCH_460011 | 1170109 | 1171209 | 1101 | *spoVE* | Factor for spore cortex peptidoglycan synthesis (stage V sporulation) |
| SSCH_460018 | 1176541 | 1178688 | 2148 | *spoVD* | Stage V sporulation protein D |
| SSCH_510003 | 1309558 | 1309995 | 438 | *_* | Sporulation protein YtaF |
| SSCH_550012 | 1440928 | 1442148 | 1221 | *_* | Sporulation integral membrane protein YlbJ |
| SSCH_630027 | 1612515 | 1613141 | 627 | *_* | Putative Stage III sporulation protein AG |
| SSCH_630029 | 1613772 | 1614944 | 1173 | *_* | Stage III sporulation protein AE |
| SSCH_630030 | 1615004 | 1615390 | 387 | *spoIIIAD* | Stage III sporulation protein AD |
| SSCH_630031 | 1615409 | 1615618 | 210 | *spoIIIAC* | Stage III sporulation protein AC |
| SSCH_630032 | 1615625 | 1616197 | 573 | *_* | Stage III sporulation protein SpoAB |
| SSCH_630033 | 1616163 | 1617146 | 984 | *spoIIIAA* | Stage III sporulation protein AA |
| **Label** | **Begin** | **End** | **Length (bp)** | **Gene** | **Product** |
| SSCH_870023 | 2075323 | 2076222 | 900 | *_* | Putative Polysaccharide deacetylase family sporulation protein PdaB |
| SSCH_1070016 | 2343438 | 2344166 | 729 | *_* | Stage II sporulation protein R |
| SSCH_1070017 | 2344177 | 2344947 | 771 | *sigG* | RNA polymerase sporulation-specific sigma factor (sigma-G) |
| SSCH_1470007 | 2785690 | 2786601 | 912 | *spoVK* | mother cell sporulation ATPase |
| SSCH_1520015 | 2836596 | 2837177 | 582 | *_* | Sporulation protein-like protein (Part 1) |
| SSCH_1520016 | 2837150 | 2837587 | 438 | *_* | Sporulation protein-like protein (Part 2) |
| SSCH_630004 | 1592650 | 1593417 | 768 | *spo0A* | Stage 0 sporulation protein A homolog |
| SSCH_700028 | 1741837 | 1742241 | 405 | *sigK* | RNA polymerase sigma-28 factor |
| SSCH_980006 | 2204582 | 2205403 | 822 | *pylD* | Pyrrolysine biosynthesis protein PylD |
| SSCH_980007 | 2205424 | 2206260 | 837 | *pylS* | Pyrrolysine--tRNA(Pyl) ligase |
| SSCH_980008 | 2206526 | 2207077 | 552 | *pylSn* | Pyrrolysine--tRNA ligase |
| SSCH_980009 | 2207180 | 2208295 | 1116 | *pylC* | 3-methylornithine--L-lysine ligase (pyrrolysine biosynthesis protein PylC) |
| SSCH_980010 | 2208295 | 2209383 | 1089 | *pylB* | 3-methylornithine synthase (Pyrrolysine biosynthesis protein PylB) |
