# Supplementary material for: Genome-Guided Analysis and Whole Transcriptome Profiling of the Mesophilic Syntrophic Acetate Oxidising Bacterium Syntrophaceticus schinkii
Source: PLoS One. 2016 Nov 16;11(11):e0166520. doi: 10.1371/journal.pone.0166520 (PMC5113046; doi:10.1371/journal.pone.0166520)
Supplement: S2 Table — (DOCX) [file pone.0166520.s013.docx]

|  | | | | | |  |  |  |  |
| --- | --- | --- | --- | --- | --- | --- | --- | --- | --- |
| **Transporter Classification (TC) Family** | | **Number of transport systems** | | | **Component proteins** |  |  |  |  |
| **I. Channels** | | |  | |  | | | | |
| 1.A.23. The Small Conductance Mechanosensitive Ion Channel (MscS) Family | | | 1 | | SSCH_970008 | | | | |
| 1.A.35. The CorA Metal Ion Transporter (MIT) Family | | | 1 | | SSCH_1320004 | | | | |
| 2.A.1.3. The Drug:H+ Antiporter-2 (14 Spanner) (DHA2) Family | | | 1 | | SSCH_520063 | | | | |
| **IIa. MFS-type permeases** | | |  | |  | | | | |
| 2.A.1.8. The Nitrate/Nitrite Porter (NNP) Family | | | 1 | | SSCH_20017 | | | | |
| 2.A.1.11. The Oxalate:Formate Antiporter (OFA) Family | | | 1 | | SSCH_800009 | | | | |
| 2.A.1.32. The Putative Aromatic Compound/Drug Exporter (ACDE) Family | | | 1 | | SSCH_1440003 | | | | |
| 2.A.1.35. The Fosmidomycin Resistance (Fsr) Family | | | 2 | | SSCH_350002,1380003 | | | | |
| **IIb. Other permeases** | | |  | |  | | | | |
| 2.A.3. The Amino Acid-Polyamine- Organocation (APC) Family | | | 2 | | SSCH_90037, 320011 | | | | |
| 2.A.4. The Cation Diffusion Facilitator (CDF) Family | | | 1 | | SSCH_1390006 | | | | |
| 2.A.3.9. The Spore Germination Protein 3 (SGP)Family | | | 1 | | SSCH_60041 | | | | |
| 2.A.6.4. The SecDF (SecDF) Family | | | 1 | | (SSCH_520074, 520073) | | | | |
| 2.A.6.2. The (Largely Gram-negative Bacterial) Hydrophobe/Amphiphile Efflux-1 (HAE1) Family | | | 1 | | (SSCH_140003, 140005) | | | | |
| 2.A.7.23. The Putative Tryptophan Efflux (Trp-E) Family | | | 1 | | SSCH_1000005 | | | | |
| 2.A.7.22. The 4 TMS Small Multidrug Resistance-2 (SMR2) Family | | | 1 | | SSCH_210001 | | | | |
| 2.A.15 The Betaine/Carnitine/Choline Transporter (BCCT) Family | | | 2 | | SSCH_450002, SSCH_450006 | | | | |
| 2.A.19. The Ca2+:Cation Antiporter (CaCA) Family | | | 1 | | SSCH_870015 | | | | |
| 2.A.21 The Solute:Sodium Symporter (SSS) Family | | | 3 | | SSCH_1190010, SSCH_330008, SSCH_1000017 | | | | |
| 2.A.9. The Cytochrome Oxidase Biogenesis (Oxa1) Family | | | 1 | | SSCH_110028 | | | | |
| 2.A.38. The K+ Transporter (Trk) Family | | | 2 | | (SSCH_1280005, 1280006, SSCH_1770011, SSCH_1770012, SSCH_770001) | | | | |
| 2.A.40. The Nucleobase:Cation Symporter (NCS2) Family | | | 1 | | SSCH_1660003 | | | | |
| 2.A.44. The Formate-Nitrite Transporter (FNT) Family | | | 1 | | SSCH_150019 | | | | |
| 2.A.47. The Divalent Anion:Na+ Symporter (DASS) Family | | | 1 | | SSCH_480010 | | | | |
| 2.A.58. The Phosphate:Na+ Symporter (PNaS) Family | | | 1 | | SSCH_350041 | | | | |
| 2.A.66.4. The Mouse Virulence Factor (MVF) Family | | | 1 | | SSCH_170009, 790020 | | | | |
| 2.A.66.1. The Multi Antimicrobial Extrusion (MATE) Family | | | 1 | | SSCH_1100010 | | | | |
| 2.A.76. The Resistance to Homoserine/ Threonine (RhtB) Family | | | 1 | | SSCH_870013 | | | | |
| 2.A.78. The Branched Chain Amino Acid Exporter (LIV-E) Family | | | 1 | | (SSCH_210014, 210015) | | | | |
| 2.A.86. The Autoinducer-2 Exporter (AI-2E) Family (Formerly the PerM Family, TC #9.B.22) | | | 1 | | SSCH_70002 | | | | |
| 2.A.96 The Acetate Uptake Transporter (AceTr) Family | | | 1 | | SSCH_800020 | | | | |
| **IIIa. ABC-type transporters** | | |  | |  | | | | |
| 3.A.1.1. The Carbohydrate Uptake Transporter-1 (CUT1) Family | | | 1 | | (SSCH_1210009, 1210007, 1210005, 1000021) | | | | |
| 3.A.1.2. The Carbohydrate Uptake (CUT2) Family | | | 2 | | [SSCH_270010, 270005, 270006 (M)] [SSCH_470005 (M), SSCH_470004 (M), SSCH_470002 (C+C)] [SSCH_470002 (C+C)] | | | | |
| 3.A.1.3. The Polar Amino Acid Uptake 1 Transporter (PAAT) Family | | | 1 | | (SSCH_350015, 350014, 350013) | | | | |
| 3.A.1.4. The Hydrophobic Amino Acid 1 Uptake Transporter (HAAT) Family | | | 1 | | [SSCH_960033 (C), SSCH_960034 (C), SSCH_960035 (M), SSCH_110002 (M), SSCH_110003 (R)] | | | | |
| 3.A.1.7. The Phosphate Uptake 1 Transporter (PhoT) Family | | | 1 | | [SSCH_80006 (M), SSCH_80005 (M), SSCH_80011, 520008, 350013 (C), SSCH_80023, 80009 (R)] | | | | |
| 3.A.1.8. The Molybdate Uptake 1 Transporter (MolT) Family | | | 1 | | [SSCH_1520005 (C), SSCH_1520006, SSCH_1520007, 680013 (M), SSCH_680012 (R)] | | | | |
| 3.A.1.12. The Quaternary Amine 1 Uptake Transporter (QAT) Family (Similar to 3.A.1.16 and 3.A.1.17) | | | 1 | | [SSCH_560023 (R), SSCH_560022 (M), SSCH_560019, SSCH_560021 (C)] | | | | |
| 3.A.1.14. The Iron Chelate Uptake 2 Transporter (FeCT) Family (Similar to 3.A.1.13 and 3.A.1.15) | | | 2 | | [SSCH_1760008 (R), SSCH_160017, 160012, 760014 (C), SSCH_160016, 160011, 730002 (M)] [SSCH_580007 (C), SSCH_580008, 250024 (M), SSCH_580006 (R)] | | | | |
| 3.A.1.15. The Manganese/Zinc/Iron Chelate Uptake Transporter (MZT) Family (Similar to 3.A.1.12, 3.A.1.14 and 3.A.1.16) | | | 1 | | [SSCH_1060012 (M), SSCH_1060013 (C), SSCH_1060014 (R)] | | | | |
| 3.A.1.16. The Nitrate/Nitrite/Cyanate 2 Uptake Transporter (NitT) Family (Similar to 3.A.1.12 and 3.A.1.17) | | | 1 | | SSCH_390016 (R)] | | | | |
| 3.A.1.17. The Taurine Uptake Transporter (TauT) Family (Similar to 3.A.1.12 and 3.A.1.16) | | | 3 | | [SSCH_1470005, 560021 (C), SSCH_1470006 (M)] [SSCH_1130013 (C)][SSCH_390016 (C), SSCH_390017 (M)] | | | | |
| 3.A.1.18. The Cobalt Uptake 1 Transporter (CoT) Family 3.A.1.23. | | | 1 | | [SSCH_790009, 870009 (C), SSCH_790008 (M), SSCH_790007 (R), SSCH_790005 (M)] | | | | |
| 3.A.1.23. The Nickel/Cobalt Uptake Transporter (NiCoT) Family (M)] | | | 3 | | [SSCH_380008 (C+C), SSCH_380010 [SSCH_790009, 1350007 (C), SSCH_1310003 (M)] [SSCH_790009, 870009 (C)] | | | | |
| 3.A.1.102. The Lipooligosaccharide Exporter (LOSE) Family | | | 1 | | [SSCH_200005, 250006, 1350025 (C), SSCH_730008 (M)] | | | | |
| 3.A.1.105. The Drug Exporter-1 (DrugE1) Family | | | 1 | | [SSCH_1220014 (C)] | | | | |
| 3.A.1.122. The Macrolide Exporter (MacB) Family | | | 1 | | [SSCH_300013 (M), SSCH_710012, 1220004, 420015 (C)] | | | | |
| 3.A.1.129. The CydDC Cysteine Exporter (CydDC-E) Family | | | 1 | | [SSCH_1350008 (M+C)] | | | | |
| Unclassified ABC-type transporters | | | 2 | | [SSCH_1220014 (C), SSCH_150007 (M)] [SSCH_1220015 (M), SSCH_1220014 (C)] | | | | |
| Orphan ABC transporter components | | | 2 | | [SSCH_1130015 (C)] [SSCH_160018, 1760011 (R)] | | | | |
| **IIIb. Other Active transporters** | | |  | |  | | | | |
| 3.A.3. The P-type ATPase (P-ATPase) Superfamily | | | 3 | | [SSCH_710032] [SSCH_620002] [SSCH_710031] | | | | |
| 3.A.10. The H+-translocating Pyrophosphatase (H+-PPase) Family IV. Phosphotransferase system | | | 1 | | [SSCH_1440001] | | | | |
| 9.A.8. The Ferrous Iron Uptake (FeoB) Family | | | 1 | | [SSCH_430004] [SSCH_680025] | | | | |
| 9.B.3. The Putative Bacterial Murein Precursor Exporter (MPE) Family | | | 2 | | [SSCH_45002] [SSCH_470040] | | | | |
| 9.B.27. The YdjX-Z (YdjX-Z) Family | | | 1 | | [SSCH_220011] | | | | |
| 9.B.31. The YqiH (YqiH) Family | | | 1 | | [SSCH_360019] | | | | |
| 9.B.32. The Putative Vectorial Glycosyl | | | 1 | | [SSCH_630001] | | |  |  |

All proteins that constitute a single transport system are enclosed in square ([ ])brackets. For the ABC-type transporters, C denotes the cytoplasmic ATPase, M is the membrane-spanning integral membrane transporter, R denotes the substrate-binding receptor, and MFP is a membrane fusion protein.
