# Supplementary material for: Genome-Guided Analysis and Whole Transcriptome Profiling of the Mesophilic Syntrophic Acetate Oxidising Bacterium Syntrophaceticus schinkii
Source: PLoS One. 2016 Nov 16;11(11):e0166520. doi: 10.1371/journal.pone.0166520 (PMC5113046; doi:10.1371/journal.pone.0166520)
Supplement: S3 Table — (DOC) [file pone.0166520.s014.doc]

| **Label** | **Begin** | **End** | **Length (bp)** | **Gene** | **Product** |
| --- | --- | --- | --- | --- | --- |
| SSCH_270011 | 707502 | 708167 | 666 | *mtbC* | Dimethylamine corrinoid protein 2 |
| SSCH_290001 | 728184 | 729512 | 1329 | *_* | Trimethylamine:corrinoid methyltransferase |
| SSCH_290002 | 729548 | 730189 | 642 | *mttC* | Trimethylamine corrinoid protein |
| SSCH_290004 | 730739 | 731722 | 984 | *_* | Trimethylamine methyltransferase |
| SSCH_290005 | 731778 | 732095 | 318 | *_* | Dimethylamine methyltransferase MtbB1 |
| SSCH_290006 | 732120 | 733169 | 1050 | *_* | Dimethylamine methyltransferase MtbB2 |
| SSCH_320004 | 799243 | 799719 | 477 | *_* | Trimethylamine:corrinoid methyltransferase |
| SSCH_320005 | 799768 | 800670 | 903 | *_* | Trimethylamine:corrinoid methyltransferase |
| SSCH_320007 | 801316 | 802302 | 987 | *_* | Trimethylamine methyltransferase |
| SSCH_320008 | 802434 | 803081 | 648 | *mttC* | Trimethylamine corrinoid protein 1 |
| SSCH_320009 | 803126 | 803446 | 321 | *_* | Dimethylamine methyltransferase MtbB2 |
| SSCH_320010 | 803465 | 804511 | 1047 | *_* | Dimethylamine methyltransferase MtbB3 |
| SSCH_330025 | 834578 | 835279 | 702 | *mtbC* | Dimethylamine corrinoid protein |
| SSCH_340003 | 837803 | 839248 | 1446 | *_* | Trimethylamine:corrinoid methyltransferase |
| SSCH_340004 | 839278 | 839904 | 627 | *mtbC* | Dimethylamine corrinoid protein 2 |
| SSCH_350017 | 864405 | 865043 | 639 | *mtbC* | Dimethylamine corrinoid protein 1 |
| SSCH_450003 | 1136990 | 1137628 | 639 | *mtbC* | Dimethylamine corrinoid protein 2 |
| SSCH_450004 | 1137696 | 1139129 | 1434 | *_* | Trimethylamine:corrinoid methyltransferase |
| SSCH_960022 | 2171889 | 2173406 | 1518 | *_* | Trimethylamine:corrinoid methyltransferase |
| SSCH_960023 | 2173435 | 2174064 | 630 | *mtbC* | Dimethylamine corrinoid protein 1 |
| SSCH_960024 | 2174096 | 2175565 | 1470 | *_* | Trimethylamine:corrinoid methyltransferase |
| SSCH_960028 | 2179151 | 2179795 | 645 | *mtbC* | Dimethylamine corrinoid protein 2 |
| SSCH_960029 | 2179811 | 2180896 | 1086 | *_* | Trimethylamine methyltransferase |
| SSCH_970001 | 2187827 | 2188135 | 309 | *_* | Dimethylamine corrinoid protein 3 |
| SSCH_1000014 | 2233820 | 2235289 | 1470 | *_* | Trimethylamine:corrinoid methyltransferase |
| SSCH_1000015 | 2235395 | 2236018 | 624 | *mtbC* | Dimethylamine corrinoid protein 1 |
| SSCH_1160005 | 2436921 | 2438384 | 1464 | *_* | Trimethylamine:corrinoid methyltransferase |
| SSCH_1160006 | 2438399 | 2439850 | 1452 | *_* | Trimethylamine:corrinoid methyltransferase |
| SSCH_1160007 | 2439924 | 2440553 | 630 | *mtbC* | Dimethylamine corrinoid protein 1 |
| SSCH_1530010 | 2842546 | 2843169 | 624 | *mttC* | Trimethylamine corrinoid protein |
| SSCH_1540002 | 2843829 | 2844131 | 303 | *_* | Trimethylamine:corrinoid methyltransferase |
| SSCH_2530004 | 3120050 | 3120676 | 627 | *mttC* | Trimethylamine corrinoid protein 1 |
| SSCH_2530005 | 3120785 | 3122149 | 1365 | *_* | Putative Trimethylamine:corrinoid methyltransferase |
