# Supplementary material for: Genome-Guided Analysis and Whole Transcriptome Profiling of the Mesophilic Syntrophic Acetate Oxidising Bacterium Syntrophaceticus schinkii
Source: PLoS One. 2016 Nov 16;11(11):e0166520. doi: 10.1371/journal.pone.0166520 (PMC5113046; doi:10.1371/journal.pone.0166520)
Supplement: S4 Table — (DOC) [file pone.0166520.s015.doc]

| **Label** | **Begin** | **End** | **Length (bp)** | **Gene** | **Product** |
| --- | --- | --- | --- | --- | --- |
| SSCH_120012 | 328210 | 329454 | 1245 | *eno* | Enolase |
| SSCH_120014 | 329563 | 331104 | 1542 | *pgm* | phosphoglycerate mutase |
| SSCH_120015 | 331101 | 331889 | 789 | *tpiA* | triosephosphate isomerase |
| SSCH_120016 | 331917 | 333047 | 1131 | *pgk* | phosphoglycerate kinase |
| SSCH_120017 | 333263 | 334270 | 1008 | *gapA* | glyceraldehyde-3-phosphate dehydrogenase |
| SSCH_120022 | 339258 | 340233 | 1026 | *-* | Glucokinase |
| SSCH_120024 | 340401 | 341774 | 1374 | *-* | Glucose-6-phosphate isomerase |
| SSCH_1210001 | 2508477 | 2509319 | 843 | *-* | fructose-1,6-bisphosphate aldolase |
| SSCH_60008 | 122534 | 124285 | 1752 | *pyk* | pyruvate kinase |
| SSCH_60009 | 124302 | 125270 | 969 | *pfkA* | 6-phosphofructokinase |
