# Supplementary material for: Genome-Guided Analysis and Whole Transcriptome Profiling of the Mesophilic Syntrophic Acetate Oxidising Bacterium Syntrophaceticus schinkii
Source: PLoS One. 2016 Nov 16;11(11):e0166520. doi: 10.1371/journal.pone.0166520 (PMC5113046; doi:10.1371/journal.pone.0166520)
Supplement: S5 Table — (DOCX) [file pone.0166520.s016.docx]

| **Locus Tag** | **Begin** | **End** | **Length** | **Gene** | **Product** |
| --- | --- | --- | --- | --- | --- |
| SSCH_20014 | 44954 | 45763 | 810 | *fdhD* | Formate dehydrogenase |
| SSCH_1520002 | 2826076 | 2827689 | 1614 | *fdhA* | Formate dehydrogenase subunit alpha |
| SSCH_1520003 | 2827702 | 2828763 | 1062 | *fdhB* | Formate dehydrogenase subunit beta |
| SSCH_370012 | 934800 | 936560 | 1761 | *fhs* | Formate-tetrahydrofolate synthetase |
| SSCH_630016 | 1604445 | 1605299 | 855 | *folD1* | Methenyl-tetrahydrofolate dehydrogenase /cyclohydrolase |
| SSCH_630017 | 1605394 | 1606263 | 870 | *folD* | Methenyl-tetrahydrofolate dehydrogenase /cyclohydrolase |
| SSCH_600032 | 1546476 | 1547417 | 942 | *metF* | Methylenetetrahydrofolate reductase |
| SSCH_600040 | 1553962 | 1555944 | 1983 | *acsA* | Bifunctional acetyl-CoA decarbonylase/synthase complex, subunit alpha |
| SSCH_860003 | 2053973 | 2054773 | 801 | *metH1* | Methyl-tetrahydrofolate:corrinoid iron-sulfur protein methyltransferase |
| SSCH_180012 | 506473 | 508338 | 1866 | *cooS* | Carbon monoxide dehydrogenase |
| SSCH_600041 | 1556211 | 1558247 | 2037 | *acsB* | Carbon monoxide dehydrogenase/acetyl-CoA synthase complex; subunit beta |
| SSCH_600042 | 1558339 | 1558575 | 237 | *cooC* | Carbon monoxide dehydrogenase accessory protein CooC |
| SSCH_550010 | 1438174 | 1439514 | 1341 | *ackA* | Acetate kinase A |
| SSCH_550011 | 1439539 | 1440594 | 1056 | *pta* | Phosphate acetyltransferase |
| SSCH_600037 | 1550747 | 1551721 | 975 | *acsD* | Corrinoid/iron-sulfur protein small subunit |
| SSCH_600038 | 1551742 | 1552485 | 744 | *acsF* | Carbon monoxide dehydrogenase nickel-insertion accessory protein |
| SSCH_600039 | 1552544 | 1553866 | 1323 | *acsC* | Corrinoid/iron-sulfur protein large subunit |
