# Supplementary material for: Genome-Guided Analysis and Whole Transcriptome Profiling of the Mesophilic Syntrophic Acetate Oxidising Bacterium Syntrophaceticus schinkii
Source: PLoS One. 2016 Nov 16;11(11):e0166520. doi: 10.1371/journal.pone.0166520 (PMC5113046; doi:10.1371/journal.pone.0166520)
Supplement: S6 Table — (DOC) [file pone.0166520.s017.doc]

| **Locus Tag** | **Begin** | | **End** | **Length (bp)** | **Gene** | **Product** | | | |
| --- | --- | --- | --- | --- | --- | --- | --- | --- | --- |
| **Putative electron-confurcating hydrogenase (Ech)** | | | | | | | | | |
| SSCH_170021 | 469306 | | 469659 | 354 | *echF* | Ech hydrogenase subunit F | | | |
| SSCH_170022 | 469688 | | 470770 | 1083 | *echE* | Ech Hydrogenase subunit E | | | |
| SSCH_170023 | 470771 | | 471148 | 378 | *echD* | Ech Hydrogenase subunit D | | | |
| SSCH_170024 | 471145 | | 471582 | 438 | *echC* | Ech hydrogenase subunit C | | | |
| SSCH_170025 | 471583 | | 472449 | 867 | *echB* | Ech hydrogenase subunit B | | | |
| SSCH_170026 | 472446 | | 474431 | 1986 | *echA* | Ech hydrogenase subunit A | | | |
| **Putative format dehydrogenases** | | | | | | | | | |
| SSCH_1520002 | 2826076 | | 2827689 | 1614 | *fdhA* | Formate dehydrogenase subunit alpha | | | |
| SSCH_1520003 | 2827702 | | 2828763 | 1062 | *fdhB* | Formate dehydrogenase subunit beta | | | |
| SSCH_1490003 | 2804689 | | 2805462 | 774 | *fdhE* | putative formate dehydrogenase formation protein | | | |
| SSCH_1490004 | 2805505 | | 2806188 | 684 | *fdhC* | Formate dehydrogenase subunit | | | |
| SSCH_1490005 | 2806194 | | 2807048 | 855 | *fdnH* | Formate dehydrogenase, nitrate-inducible, iron-sulfur subunit | | | |
| SSCH_1490006 | 2807018 | | 2808535 | 1518 | *fdhA* | Formate dehydrogenase, alpha subunit | | | |
| **Putative (electron-bifurcating) ferredoxin/NADH dependent [Fe-Fe] hydrogenases** | | | | | | | | | |
| SSCH_210008 | 590534 | | 592258 | 1725 | *hndD* | NADH-reducing hydrogenase subunit HndC | | | |
| SSCH_210009 | 592264 | | 593484 | 1221 | *hndC* | NADH-reducing hydrogenase subunit HndC | | | |
| SSCH_210010 | 593510 | | 593953 | 444 | *hndA* | NADH-reducing hydrogenase subunit HndA | | | |
| SSCH_90017 | 225580 | | 227373 | 1794 | *hndC* | NADH-reducing hydrogenase subunit HndC | | | |
| SSCH_90018 | 227405 | | 227776 | 372 | *hndB* | NADH-reducing hydrogenase subunit HndB | | | |
| SSCH_90019 | 227875 | | 228366 | 492 | *hndA* | NADH-reducing hydrogenase subunit HndA | | | |
| SSCH_1120014 | 2387250 | | 2389064 | 1815 | *hndC* | NADH-reducing hydrogenase subunit HndC | | | |
| SSCH_1120015 | 2389061 | | 2389555 | 495 | *hndA* | NADH-reducing hydrogenase subunit HndA | | | |
| SSCH_600009 | 1528791 | | 1529438 | 648 | *nuoG3* | NADH-quinone oxidoreductase subunit | | | |
| SSCH_600010 | 1529428 | | 1531338 | 1911 | *nuoF4* | NADH-quinone oxidoreductase 51 | | | |
| SSCH_600011 | 1531335 | | 1531868 | 534 | *ndufv2* | NADH dehydrogenase [ubiquinone] flavoprotein | | | |
| **Putative periplasmic [Ni-Fe] hydrogenase** | | | | | | | | | |
| SSCH_30031 | 75215 | | 76294 | 1080 | *hydA* | Periplasmic [NiFeSe] hydrogenase small subunit | | | |
| SSCH_30032 | 76275 | | 77693 | 1419 | *hydB* | Periplasmic [NiFeSe] hydrogenase large subunit | | | |
| SSCH_30033 | 77706 | | 78362 | 657 | *hydC* | Quinone-reactive Ni/Fe-hydrogenase b-type cytochrome subunit | | | |
| **Putative cytoplasmic [Ni-Fe] hydrogenase** | | | | | | | | | |
| SSCH_370002 | 924012 | | 924776 | 765 | _ | NADH ubiquinone oxidoreductase 20 kDa subunit | | | |
| SSCH_370003 | 924773 | | 926086 | 1314 | *hydA* | Nickel-dependent hydrogenase large subunit | | | |
| SSCH_370004 | 926087 | | 926554 | 468 | *hydD* | Hydrogenase 1 maturation protease | | | |
| SSCH_370005 | 926876 | | 927217 | 342 | *hypA* | hydrogenase nickel incorporation protein HypA | | | |
| SSCH_370006 | 927223 | | 927879 | 657 | *hypB* | Hydrogenase accessory protein HypB | | | |
| **Hydrogenases maturation proteins** | | | | | | | | | |
| SSCH_60028 | 141154 | | 142176 | 1023 | *hypE* | Carbamoyl phosphate phosphatase, hydrogenase 3 maturation protein | | | |
| SSCH_60029 | 142173 | | 143273 | 1101 | *hypD* | Hydrogenases maturation protein | | | |
| SSCH_60030 | 143251 | | 143472 | 222 | *hypC* | Hydrogenase expression/formation protein | | | |
| **Putative NADH oxidoreductase/heterodisulfide reductase complex** | | | | | | | | | |
| SSCH_160001 | 431517 | | 432416 | 900 | *hdrB* | CoB--CoM heterodisulfide reductase 1 subunit B | | | |
| SSCH_160002 | 432433 | | 433017 | 585 | *hdrA2* | CoB--CoM heterodisulfide reductase iron-sulfur subunit A 2 | | | |
| SSCH_160003 | 433033 | | 434409 | 1377 | *hrdA* | CoB--CoM heterodisulfide reductase iron-sulfur subunit A | | | |
| SSCH_160004 | 434400 | | 434597 | 198 | *frhD* | Coenzyme F420-reducing hydrogenase, delta subunit | | | |
| SSCH_160005 | 434658 | | 434819 | 162 | *flpD* | Methyl-viologen-reducing hydrogenase, delta subunit | | | |
| SSCH_160006 | 434812 | | 435702 | 891 | *RnfC* | 4Fe-4S ferredoxin, iron sulfur binding domain | | | |
| SSCH_160007 | 435730 | | 436734 | 1005 | *asrA* | 4Fe-4S ferredoxin, iron sulfur binding domain | | | |
| SSCH_160008 | 436727 | | 437554 | 828 | *asrB* | NAD(P)-binding oxidoreductase B | | | |
| **RNF complex** | | | | | | | | | |
| SSCH_420047 | 1057496 | | 1058332 | 837 | *rnfB* | Electron transport complex protein RnfB | | | |
| SSCH_420048 | 1058354 | | 1058932 | 579 | *rnfA* | Electron transport complex protein RnfA | | | |
| SSCH_420049 | 1058929 | | 1059549 | 621 | *rnfE* | Electron transport complex protein RnfE | | | |
| SSCH_420051 | 1059551 | | 1059748 | 198 | *rnfG* | Electron transport complex protein RnfG | | | |
| SSCH_420052 | 1060115 | | 1061122 | 1008 | *rnfD* | Electron transport complex protein RnfD | | | |
| SSCH_420053 | 1061127 | | 1062323 | 1197 | *rnfC* | Electron transport complex protein RnfC | | | |
|  | |  | |  | |  |  |  |  |
|  | |  | |  | |  |  |  |  |
